# Classification of ROI-based fMRI data in short-term memory tasks using discriminant analysis and neural networks

**DOI:** 10.3389/fninf.2024.1480366

**Published:** 2024-12-20

**Authors:** Magdalena Fafrowicz, Marcin Tutajewski, Igor Sieradzki, Jeremi K. Ochab, Anna Ceglarek-Sroka, Koryna Lewandowska, Tadeusz Marek, Barbara Sikora-Wachowicz, Igor T. Podolak, Paweł Oświęcimka

**Affiliations:** ^1^Department of Cognitive Neuroscience and Neuroergonomics, Jagiellonian University, Kraków, Poland; ^2^Institute of Theoretical Physics, Jagiellonian University, Kraków, Poland; ^3^Group of Machine Learning Methods GMUM, Faculty of Mathematics and Computer Science, Jagiellonian University, Kraków, Poland; ^4^Mark Kac Center for Complex Systems Research, Jagiellonian University, Kraków, Poland; ^5^Faculty of Psychology, SWPS University, Katowice, Poland; ^6^Complex Systems Theory Department, Institute of Nuclear Physics, Polish Academy of Sciences, Kraków, Poland

**Keywords:** explainability, fMRI, working memory, ROI, machine learning, neural network

## Abstract

Understanding brain function relies on identifying spatiotemporal patterns in brain activity. In recent years, machine learning methods have been widely used to detect connections between regions of interest (ROIs) involved in cognitive functions, as measured by the fMRI technique. However, it's essential to match the type of learning method to the problem type, and extracting the information about the most important ROI connections might be challenging. In this contribution, we used machine learning techniques to classify tasks in a working memory experiment and identify the brain areas involved in processing information. We employed classical discriminators and neural networks (convolutional and residual) to differentiate between brain responses to distinct types of visual stimuli (visuospatial and verbal) and different phases of the experiment (information encoding and retrieval). The best performance was achieved by the LGBM classifier with 1-time point input data during memory retrieval and a convolutional neural network during the encoding phase. Additionally, we developed an algorithm that took into account feature correlations to estimate the most important brain regions for the model's accuracy. Our findings suggest that from the perspective of considered models, brain signals related to the resting state have a similar degree of complexity to those related to the encoding phase, which does not improve the model's accuracy. However, during the retrieval phase, the signals were easily distinguished from the resting state, indicating their different structure. The study identified brain regions that are crucial for processing information in working memory, as well as the differences in the dynamics of encoding and retrieval processes. Furthermore, our findings indicate spatiotemporal distinctions related to these processes. The analysis confirmed the importance of the basal ganglia in processing information during the retrieval phase. The presented results reveal the benefits of applying machine learning algorithms to investigate working memory dynamics.

## 1 Introduction

Working memory (WM) is crucial for preparing and organizing goal-directed behaviors, with its functions of storing and manipulating incoming information. This process is capacity-limited, demanding a balance between stability (preserving the WM content from irrelevant information) and flexibility (updating WM with relevant information) (Trutti et al., 2021). It involves three temporal subprocesses: encoding, maintenance, and retrieval. Over the years, various models of WM functioning have been created, but the new ones are based more on the dynamics of the ongoing processes. The dynamic-processing model of working memory (Rose, 2020) assumes that relevant information is retained by creating representations through recurrent activity and/or strengthening the synaptic weights between neurons. These processes are very dynamic and context-dependent. One of the leading hypotheses in functional neuroimaging studies on false memory retrieval is the sensory reactivation hypothesis. It suggests that memory retrieval reactivates the processes from the encoding stage, with true memories involving activation of sensory areas (see Abe, 2012). However, recent findings indicate that content representations during retrieval differ from those during encoding. Memory retrieval appears to be more of a constructive and dynamic process involving the frontoparietal cortex, rather than just the reactivation of the sensory cortex as suggested by the sensory reactivation hypothesis (see Favila et al., 2020 for a review). To investigate the dynamic of working memory processes, we designed an fMRI experiment with four visual working memory tasks: two with visuospatial and two with verbal stimuli based on the Deese–Roediger–McDermott (DRM) paradigm (Deese, 1959; Roediger and McDermott, 1995), and additionally resting-state procedure. The DRM paradigm is widely used in memory research, as it separates the WM subprocesses like encoding and retrieval.

In recent years, a variety of innovative methods have been applied to the analysis of fMRI data. These include machine learning algorithms, nonlinear time series analysis, and complex network methodologies (Ochab et al., 2022; Singh et al., 2022; Wen et al., 2018; Onias et al., 2014). Significant research has focused on utilizing machine learning techniques and neural networks for fMRI data analysis. The main idea behind this approach is to analyze the neuroimaging data not from the point of view of the single voxel, but by identifying the patterns of neural activity over many brain areas. Thus, classification based on advanced computational algorithms is one of the most efficient methods for identifying neural activity and extracting the complex relationship between the experimental conditions and spatial-temporal patterns of brain responses measured by the fMRI technique (O'Toole et al., 2007). However, the machine learning algorithms and neural network types must be matched to the problem investigated to obtain statistically reasonable results, which is not a trivial problem due to the variety of computational methods, e.g., linear discriminant analysis (LDA), support vector machines (SVM), random forests (RF), neural networks classifiers and many others. Therefore, the performance of machine learning algorithms has been examined in many neuroscience studies, including research related to the classification and diagnosis of Alzheimer's, Huntington and schizophrenia disease (Sarraf and Tofighi, 2016; Patel et al., 2016), cognitive functions (Wen et al., 2018), sleep studies (Li et al., 2018b) and conscious visual perceptions (Haynes and Rees, 2005).

The goal of this paper is twofold. First, we would like to apply some of the most commonly used machine learning algorithms and neural networks in the study with working memory and verify their effectiveness in classifying the tasks (recognition between the visuospatial and verbal stimuli) and phases of the experiments (encoding and retrieval). In this study, we considered a set of linear and nonlinear classifiers and a residual neural network. We also regarded two kinds of data organizations, i.e. in the temporal-spatial form and single-time points from each observation. Secondly, based on the results from classification experiments, we would like to determine the brain regions that are the most important for classifiers; thus, important from the point of view of information processing in the brain. To this end, we proposed a novel algorithm and compared the results with the outcomes from the literature.

## 2 Experiment and data

### 2.1 Data description

Functional magnetic resonance imaging (fMRI) data from four short-term memory tasks and a resting-state procedure were analyzed. Based on questionnaires and genotyping of the PER3 gene, 66 participants out of 5,354 volunteers were selected to perform the tasks in the MR scanner during two sessions: morning and evening. After further data quality control, 58 participants were included in the analysis. The order of the sessions as well as the versions of the tasks (there were two equivalent versions of each task) were counterbalanced between participants. The experiment was conducted on one day (if the morning session was the first) or two days (if the evening session was the first). Participants spent the night before or between sessions in the room located in the laboratory, and their quality of sleep during that night and the week preceding the experiment was controlled using actigraphs.

The short-term memory tasks were based on Deese-Roediger-McDermott (DRM) paradigm (Deese, 1959; Roediger and McDermott, 1995), which allows separating the encoding and retrieval processes, and is dedicated to studying short-term memory distortions. Two tasks evaluated the perceptual similarity (focusing on global, GLO, and local, LOC, information processing of abstract objects), and the remaining two the verbal similarity (semantic, SEM, and phonological, PHO, task). More specifically, in GLO task, the stimuli were abstract figures requiring holistic processing; in LOC task, the objects required local processing and differed in one specific detail. On the other hand, in SEM task, participants had to remember four Polish words matched by semantic similarity, and in PHO task, matched by phonological similarity.

In each task, the goal of a participant was to memorize the memory set (encoding phase, 1.2–1.8 s), and then (after a mask or distractor) recognize if the currently displayed stimulus, called probe, was present in the preceding set (retrieval phase, 2 s). There were three possible conditions of the probe: positive (the stimulus was the same as presented in the encoding phase), negative (the stimulus was completely different) or a lure (the stimulus resembled those presented in the memory set). Participants were asked to answer with the right-hand key for “yes” and the left-hand key for “no” responses. Then, after intertrial interval (on average 8.4 s), a fixation point (450 ms) and a blank screen (100 ms), the new memory set was presented on the screen. Each task had 60 sets of stimuli followed by 25 positive probes, 25 lures, and 10 negative probes. The examples of experimental tasks are depicted in Supplementary Figure 1.

In the resting-state procedure (REST), participants were instructed to lie in the scanner with their eyes open and not to think about anything in particular. They were not involved in any cognitive process. Participants' awakeness was monitored using an eye-tracking system (Eyelink 1000, SR research, Mississauga, ON, Canada).

Structural and functional data were collected on a 3T scanner Skyra (Siemens Magnetom, Erlangen, Germany) in Małopolska Center of Biotechnology in Kraków, Poland, with a 64-channel head coil. For tasks, 709 volumes (for GLO, LOC, and SEM), and 736 volumes (for PHO task) with a T2*-weighted echo-planar sequence were acquired. For the resting-state procedure, 335 volumes with a gradient-echo single-short echo planar imaging sequence were acquired. The following scanning parameters were used: TR = 1,800 ms, TE = 27 ms, flip angle = 75°, FOV = 256 mm, voxel size: 4 × 4 × 4 mm). Structural data were acquired for each participant using a T1-weighted MPRAGE sequence with isotropic voxels (1 × 1 × 1.1 mm) using the following parameters: 256 × 256 mm matrix, 192 slices, TR = 2,300 ms, TE = 2.98 ms. Stimuli were projected on a screen behind a participant's head. The participants viewed the screen in a 45° mirror fixated on the top of the head coil.

### 2.2 Data preprocessing

**Figure 1:**
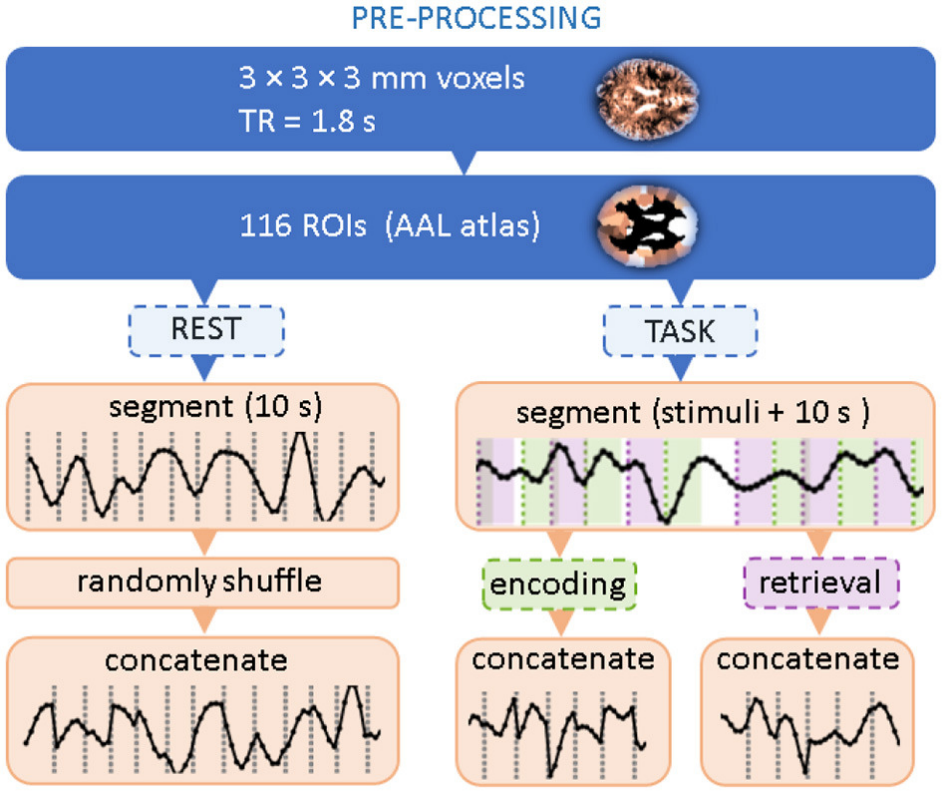
Flow chart of data preprocessing. Two topmost steps correspond to standard BOLD signal processing and coarse-graining into atlas-based Regions of Interest. The dashed boxes indicate that only a part of the experimental time series is selected.

The flow chart in Figure 1 summarizes data preprocessing steps. Following the paper that introduced the dataset (Fafrowicz et al., 2019), we performed the preprocessing using the Statistical Parametric Mapping software package (SPM12, Welcome Department of Imaging Neuroscience, UCL, London, United Kingdom) implemented in MATLAB (Mathworks, Inc., MA, United States). Scans were slice-time corrected, realigned by inclusion of field maps, co-registered, and normalized to the EPI template in Montreal Neurological Institute (MNI) stereotactic space with a voxel resolution 3 × 3 × 3 mm. Then, data were spatially smoothed using a Gaussian kernel of FWHM 6 mm, covariates like motion parameters, mean signal, white matter, and CSF were removed by linear regression. The signal was then filtered with a 0.01–0.1 Hz filter, detrended, and despiked.

The signals were then averaged within 116 brain Regions of Interest (ROIs) using the Automated Anatomical Labeling (AAL1) brain atlas (Tzourio-Mazoyer et al., 2002). For a given experimental session, they formed a data matrix with 116 rows corresponding to the ROIs and the number of columns corresponding to the length of the time series. Further processing steps follow (Ochab et al., 2022): for each session, we extracted all data segments (i.e., columns) related to the encoding or retrieval phase; for each phase, the segments were then concatenated in order of appearance. The segments started at the stimulus onset and were 10s long (6–7 TRs) each, which means that the encoding segments encompassed the presentation of the distractor and the retrieval segments encompassed the inter-trial interval. For a given session, the concatenation of segments from all 60 stimuli resulted in a 400-TR-long time series. Data for the resting state with eyes open were preprocessed similarly to the task data. First, in the absence of stimuli that would set the position of the segments, 400-TR long series were randomly chosen. Then, they were divided into consecutive, non-overlapping segments of 10s. Then, these segments were randomly shuffled and concatenated.

### 2.3 Classification tasks

We create eight classification problems using the data described in Section 2.1, four for each of the two phases of *encoding* (ENC) and *retrieval* (RET). Their variants are binary or 4-class problems depending on whether we group similar stimuli or not, e.g. *global* and *local* stimuli are both graphical and can be grouped into a single class. They can be further complicated by adding the resting state as an additional class, resulting in a 3- or 5-class classification. Table 1 lists the resulting experimental setups.

**Table 1:** Classification of the tasks due to the number of classes.

**Experiment name**	**Class 1**	**Class 2**	**Class 3**	**Class 4**	**Class 5**
ENC2	GLO, LOC	SEM, PHO			
ENC3	GLO, LOC	SEM, PHO	REST		
ENC4	GLO	LOC	SEM	PHO	
ENC5	GLO	LOC	SEM	PHO	REST
RET2	GLO, LOC	SEM, PHO			
RET3	GLO, LOC	SEM, PHO	REST		
RET4	GLO	LOC	SEM	PHO	
RET5	GLO	LOC	SEM	PHO	REST

For each of the listed setups, we randomly split the available data into training and testing subsets using a ratio of 90:10.

In all non-neural classifiers, a sample of input data always has the dimensions of 116 × 1 (number of brain regions times one time point). We also conduct additional experiments taking into account the time dimension of the data, where we train neural networks on samples of dimensions 116 × 6 (sequences of six time points). We elaborate on this experiment in Section 5.2.

## 3 Models

We have conducted the fMRI classification by dividing machine learning methods into classical linear and non-linear discriminators, and neural networks. In total, ten classifiers were compared in the study.

### 3.1 Linear classifiers

#### 3.1.1 Ridge classifier

Ridge classifier was proposed in Hoerl and Kennard (1970). The method addresses the problem of parameter estimation for multi-collinear independent variables. Ridge achieves that by adding a penalty term L2, which is equal to the square of the coefficients. However, while the L2 regularization minimizes coefficients, it never reduces them to zero (Hoerl and Kennard, 1970).

#### 3.1.2 Logistic regression

Logistic regression is another linear estimation algorithm that was tested in the study. Unlike the Ridge Classifier, the logistic regression uses a cross-entropy loss function to output the probability for the classification (Brown and Mues, 2012).

#### 3.1.3 Support vector machines using stochastic gradient descent (SGD)

Loss optimization in linear models can also be performed using stochastic gradient descent SGD. It attempts to discover the gradients of the cost function for a random selection of data points. By conducting this operation, stochastic gradient descent can lead to much faster convergence of the algorithm. This timing advantage is crucial in the case of Support Vector Machines, which tend to build complex hyperplanes (Wang et al., 2012).

#### 3.1.4 Gaussian naive Bayes

Gaussian naive Bayes is a subset of the Naive Bayes models. The model assumes a Gaussian distribution of the data and a lack of feature dependencies within it. Due to its simplicity, the classifier often performs well on highly dimensional data. It can converge faster than discriminative algorithms such as Random Forest or Logistic Regression. The Gaussian Naive Bayes can also serve as a baseline due to its probabilistic nature (Brown and Mues, 2012; Jahromi and Taheri, 2017).

#### 3.1.5 Linear discriminant analysis (LDA)

LDA is another linear method that was evaluated in the study. LDA operates in two stages. First, it extracts all the feature values linearly. Then it uses those mappings and attempts to linearly separate classes by presenting points of opposite classes as far as possible from each other (Wu et al., 1996).

### 3.2 Non-linear classifiers

In contrast to the discriminators utilizing linear functions, non-linear classifiers attempt to match the data by minimizing functions that do not share regular slopes. This approach allows for creating more strict boundaries between classified data points, hence increasing the goodness of a fit. However, at the same time, this can lead to non-linear classifiers overfitting.

#### 3.2.1 Quadratic discriminant analysis (QDA)

Unlike LDA, which relies on the assumption of linear divisibility of the feature values, Quadratic Discriminant Analysis, uses non-linear attributes to separate data points. This proves to be generally more accurate, especially in the problems with high dimensionality (Wu et al., 1996).

#### 3.2.2 Random forest classifier

Random forest classifier is a model composed of a collection of decision trees. In simple terms, decision trees can accept both numeric and categorical inputs to build sets of rules. Those rules are distributed throughout the trees. The data input can then flow in a top-to-bottom way and be filtered to produce respective outputs and classification results. Random forests use sets of such rules to provide even more accurate classification boundaries (Brown and Mues, 2012; Anyanwu and Shiva, 2009).

#### 3.2.3 Light gradient boosting machine (LGBM)

Over the last few years, boosting methods have been receiving more attention as their performance shows improvement over simple tree-based approaches. The reason for this phenomenon originates in the architecture of the boosted models. Boosting combines multiple models that are built iteratively, rather than in parallel. This ensures that each consecutively built algorithm attempts to minimize the errors made by previous models (Yaman and Subasi, 2019). Light gradient boosting is a special case of such an application, as it proves to outperform most of the other machine learning algorithms in a variety of benchmarks (Ke et al., 2017).

### 3.3 Performance baseline

#### 3.3.1 Dummy classifier

To assess the reliability of the performed experiments, We created a dummy classifier whose sole purpose was to create a baseline for the other methods. The dummy classifier was fit in such a way that it ignored the input features and relied only on the distribution of the classified classes in making predictions.

### 3.4 Neural networks

One of the motivations for using deep neural networks, specifically the ones employing convolutional layers (like convolutional and residual networks), is the ability to process spatial data. In our case, the original BOLD data can be considered as having two dimensions—the first corresponds to the spatial distribution of the ROI, and the second corresponds to the temporal character of the time series. Each data sample consists of up to six discrete measurements, one TR apart, corresponding to the duration of the encoding/retrieval phases. We hypothesize that having the data in the temporal-spatial form and a model processing this data structure can yield better results than using single-time observations.

#### 3.4.1 Layered convolutional network (CNN)

The 1D convolution is often used for processing temporal data due to its ability to effectively handle sequential information in a channel-wise manner (Kiranyaz et al., 2019; Bai et al., 2018). The CNN architecture we use comprises two 1D convolutional layers (all the convolutional layers use filters of size 3), 1D maximal pooling, another 1D convolution, a linear layer with dropout, and the final softmax layer to calculate class probabilities. Crucially, all the convolutions move along the temporal dimension. This setup allows interactions between any subset of brain regions.

#### 3.4.2 Residual network (ResNet)

**Figure 2:**
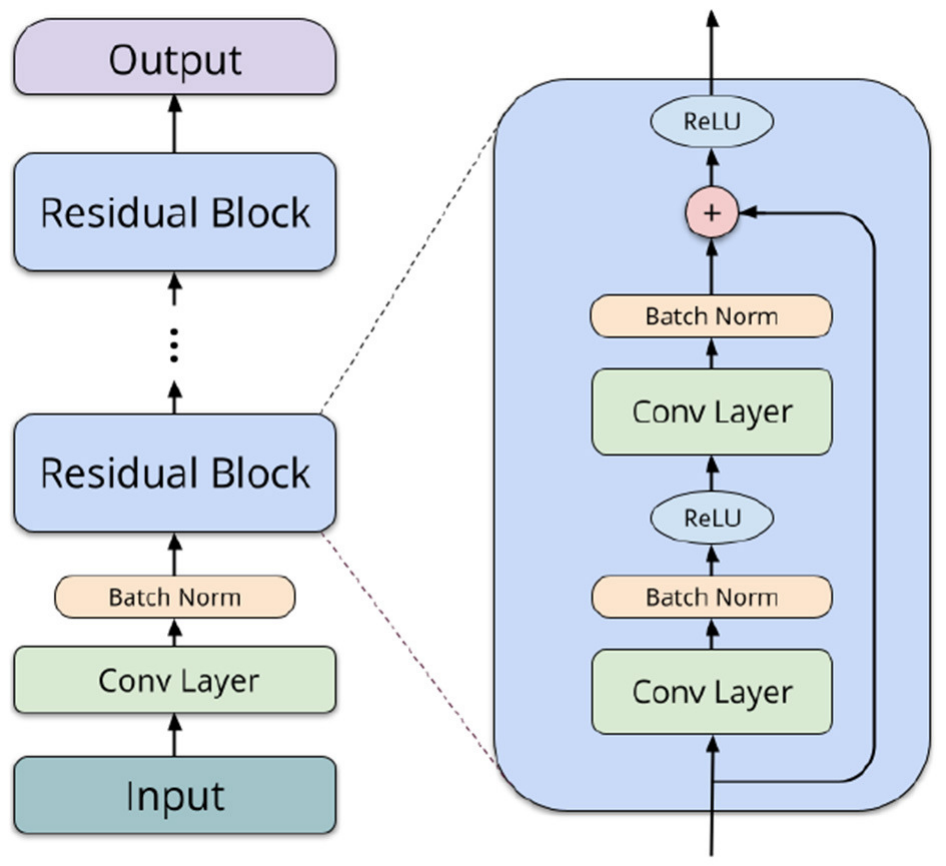
Schematic overview of a residual network block.

Residual networks (ResNets) (He et al., 2016) are a more powerful neural network type, in our case also utilizing convolutions. They consist of an input convolution, followed by several *residual blocks* of layers, depending on the network depth. Each of the blocks comprises two convolutional operations (3 × 3 filters), batch normalization (Ioffe and Szegedy, 2015), and a shortcut connection (1 × 1 filters). In the final layers, average pooling is performed, and a linear operation computes logits for each label. We utilize 2D convolutions to capture both spatial and temporal interactions between different ROIs. Taking inspiration from Li et al. (2018a) and Suk et al. (2017), we organize the ROIs in a structured format within a 2D matrix. This enables our network to effectively learn interactions between brain regions neighboring in the AAL atlas (typically, spatially adjacent or contralateral), allowing for the extraction of both local spatial relationships and temporal patterns simultaneously. Figure 2 shows a schematic overview of a typical architecture of a residual network.

## 4 Methods

This section expands on two out of the several models described in Section 3: LGBM and ResNet. This selection was made after the initial results, where LGBM and ResNet models proved to be the most promising ones (LGBM scores were comparable or higher than all other models, but for ResNet in two encoding experiments). Consequently, we included here the hyperparameter tuning and feature explanation procedures of the two models only.

### 4.1 Model training and tuning

#### 4.1.1 LGBM hyperparameter tuning

In our preliminary calculations, the tested machine learning algorithms were capable of resolving the complexity of correlations and differences within our sample of the fMRI data with varying accuracy. Therefore, we decided to pick the most promising model—LGBM—and improve it with hyperparameter tuning (Feurer and Hutter, 2019).

To perform the parameter searches, we used Optuna, a state-of-the-art hyperparameter optimization framework (Akiba et al., 2019). We conducted 100 repetitions of each experiment, such as ENC2 or RET4. Within each repetition, there were 100 hyperparameter searches, and optional pruning of trials with unpromising initial results. This procedure reduces the bias of human-produced parameters and can be utilized again, even if the data distributions have changed (Akiba et al., 2019). The parameters used for the experiments can be seen in Table 2. Henceforth, we refer to the hyperparameter-tuned Light Gradient Boosting model as “tuned LGBM.”

**Table 2:** LGBM hyperparameter tuning values.

**Hyperparameters**	**Values**
Lambda l1	1e-8 to 10
Lambda l2	1e-8 to 10
No. leaves	2 to 128
Feature fraction	0.1–1.0
Bagging fraction	0.1–1.0
Bagging freq	1–7

#### 4.1.2 Neural network training and hyperparameter tuning

In neural network experiments, each model is trained from scratch for up to 100 epochs. Early stopping was employed to finish the training process before epoch 100 if no improvement in validation loss was recorded for five epochs in a row. All networks were trained using the Adam optimizer (Kingma and Ba, 2014) together with a scheduler to reduce learning rate on loss function plateaus.

We perform a hyperparameter search for each task, where each task deals with a different data collection phase (either *encoding* or *retrieval*) and a varying number of classes—depending on whether the labels are simplified, by joining similar stimuli, and the addition of *resting state* data. Table 3 lists hyperparameters and their value ranges searched during training for ResNets and, similarly, Table 4 lists the hyperparameters for the 1D convolutional model.

**Table 3:** Residual network hyperparameter tuning values.

**Hyperparameters**	**Values**
ResNet size	14, 20, 32, 56
Batch size	32, 64, 128, 256
Learning rate	0.0001, 0.0005, 0.001, 0.005, 0.01
Dropout	0.0, 0.1, 0.2, 0.3
Weight decay	0.01, 0.05, 0.1

**Table 4:** 1D CNN hyperparameter tuning values.

**Hyperparameters**	**Values**
Batch size	32
Learning rate	0.0001, 0.0005, 0.001
Dropout	0.0, 0.1, 0.2, 0.3

### 4.2 Algorithms for data importance extraction

#### 4.2.1 ROI importance estimation for LGBM

In addition to obtaining classification metrics for the experiments carried out, we were also interested in determining which particular ROIs were the most vital features for the classifier. To this end, we extracted ROI importance scores from the LGBM models using the default LGBM feature importance estimation method (Ke et al., 2017), i.e. the number of splits. The method, applicable to any tree-based model, counts the number of times a given feature (ROI activations) was used to split the data to grow the decision tree. We report the results of the tuned LGBM in the Supplementary material.

**Figure 3:**
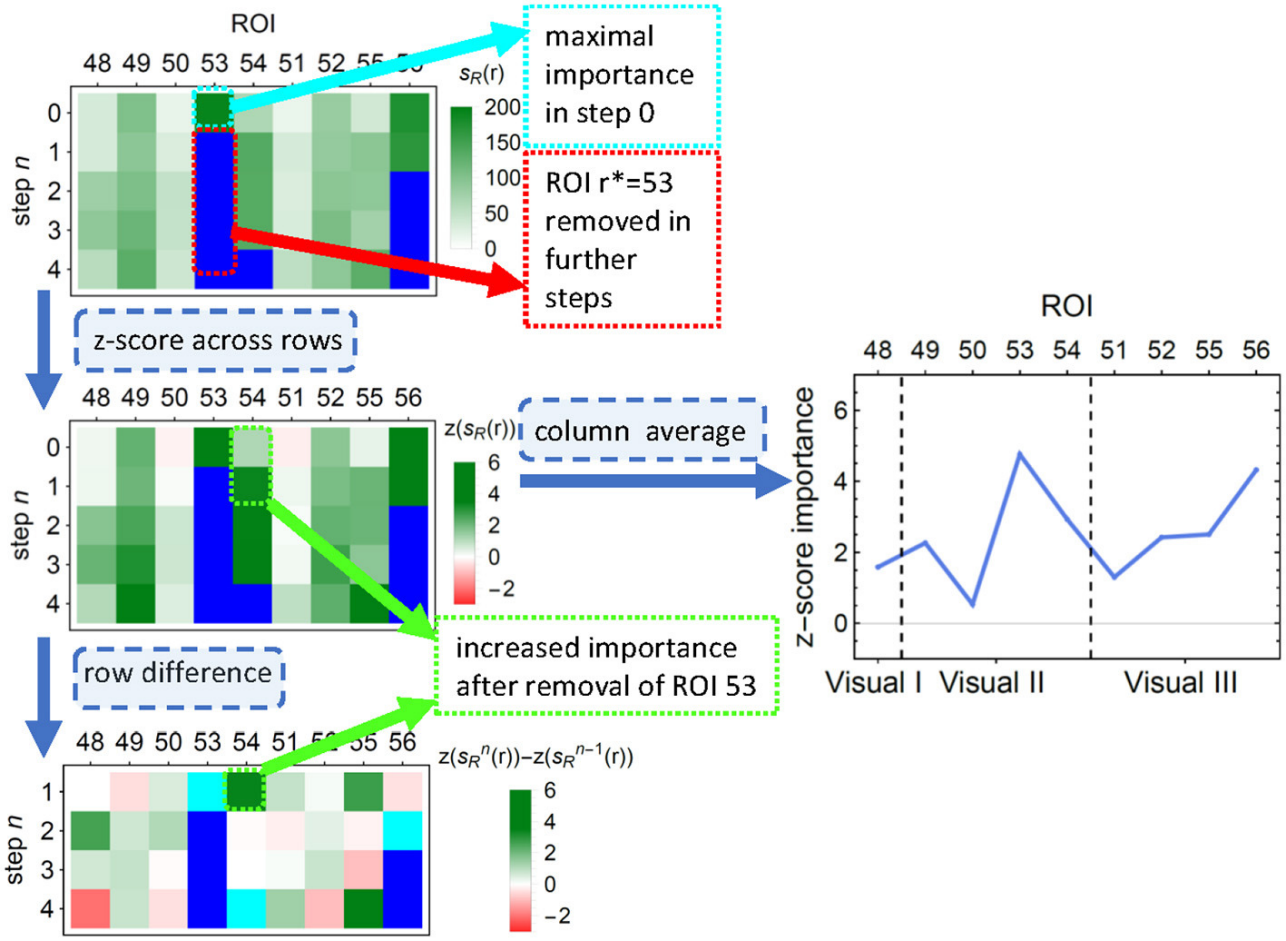
An illustration of the first few steps of Algorithm 1 on a subsample of LGBM classification results. **(Top)** Each row depicts the LGBM split scores. In consecutive steps, ROIs number 53, 56, 81 (not shown), and 54 are removed, and the scores are recalculated. **(Middle left)** Importance values are *z*-scored to correct for the decreasing number of features. **(Middle right)** The average across all removal iterations forms the final importance score of an ROI. ROIs are ordered into functionally associated groups of brain regions. **(Bottom)** The differences between importance scores indicate how classification is affected by feature correlations. Cyan (blue) pixels—regions removed in a given step *n* (in a previous step); green (red) pixels—regions whose importance increased (decreased) due to the removal. This diagram corresponds to the upper right corner in the top panel of Figure 7.

Unfortunately, such scores—as many others (see, e.g. Molnar, 2020, Ch. 8.1.4, 9.1.3, 9.5)—cannot account for possible correlations between the features. This lack could result, for example, in a pair of equally informative and highly correlated features being recognized as an important and an unimportant one; if, however, the former was removed from the data, the latter would take over its importance. To alleviate this problem, we performed an additional feature pruning procedure as described in the Algorithm 1: at each step, (i) the most important feature was removed from the training set, (ii) importance scores of the remaining features were recomputed, (iii) to correct for the decreasing number of features, the mean of importance across the remaining features was subtracted from the importance of each individual feature. The process finished when all features have been removed. To obtain the final corrected score, such demeaned scores were averaged over all the steps in which ROI was present in the model. The process is illustrated in Figure 3. Given the significant computational time and resources required for feature pruning, we limited the validation to untuned LGBM models.

**Algorithm 1:** Importance algorithm with ROI pruning.

Require: *X*—train set
Require: C—classifier
Require: *s*_*R*_(*r*)—importance scoring function for ROIs *r* ∈ *R*
Require: *S*—map for scores for each ROI
*R*←ROIs
*X*_*R*_←*X*
while |*R*| != 0 **do**
$C^{*}\leftarrow C$ trained on the set *X*_*R*_ ⊳ Train the classifier
Calculate importance score *s*_*R*_(*r*) for $C^{*}$
*s*_*R*_(*r*)←*z*(*s*_*R*_(*r*)) ⊳ Standardize (z-score)
Append *s*_*R*_(*r*) to *S*[*r*] for each *r* ∈ *R*
$r^{*}={\mathrm{argmax}}_{r\in R}s_{R}\left(r\right)$ ⊳ Find and remove the most important ROI
*R* ← *R*\{r^*^}
$X_{R}\leftarrow X_{R}\text{without row }r^{*}$
end **while**
*r*←0
while *r*<|ROIs| **do** ⊳ Calculate average scores
*S*[*r*]←median(*S*[*r*])
*r* ← *r*+1
end **while**

#### 4.2.2 ROI importance estimation for ResNets

By design, deep neural networks are black-box models, i.e., there is no simple way to obtain insight about why they generated a certain prediction. As opposed to a linear model, in deep neural networks the input representation is non-linearly transformed multiple times. Because of that, to extract interpretable knowledge about a network's decision process, we have to resort to various heuristics.

Our approach, outlined in Algorithm 2, is inspired by methods that perturb the input data during evaluation and measure the decrease in performance (Ribeiro et al., 2016). More formally, given a trained fixed ResNet model *f* and a validation set *X* = (*x*_1_, *x*_2_, …, *x*_*n*_), we sample batches from the validation set, *X*_*B*_ = (*x*_*B*1_, …, *x*_*Bk*_) and perturb in them a subset *R* of ROIs ${\tilde{X}}_{B}=P\left(X_{B},R\right)$. Both the perturbed batch ${\tilde{X}}_{B}$ and the original *X*_*B*_ are then passed to the model and have their predictions compared. Finally, differing predictions contribute to the scores collected for each perturbed ROI in *R*.

**Algorithm 2:** Importance algorithm with data perturbation.

Require: *f*—classification model
Require: *N*—number of iterations
Require: *X*—validation set
Require: *k*—ROI subset size
Require: *P*(*x, R*)—perturbation function
Require: *S*[·]—map for scores buffers for each *r* ∈ ROI
*i*←0
while *i* < *N* **do**
Sample uniformly subset *R*⊂ROI of size *k* = |*R*|
for *X*_*B*_ ∈ *X* **do**
Perturb input data ${\tilde{X}}_{B}\leftarrow P_{\emptyset }\left(X_{B},R\right)$
Forward both batches through the network $p_{B}\leftarrow f\left(X_{B}\right),{\tilde{p}}_{B}\leftarrow f\left({\tilde{X}}_{B}\right)$
Calculate importance score $s\leftarrow \frac{1}{\left|X_{B}\right|}\underset{x\in X_{B}}{\sum }1_{p\neq \tilde{p}}$
Store scores for each *r* ∈ *R* to the buffer *S*[*r*]←*S*[*r*]∪*s*
end **for**
*i* ← *i*+1
end **while**
*r*←0
while *r* < |ROIs| **do** ⊳ Calculate average scores
*i*←|*S*[*r*]|
$S\left[r\right]=\frac{1}{i}\sum S\left[r\right]$
*r* ← *r*+1
end **while**

In more detail, the perturbation function *P*(·, *R*) is assumed to change only a subset of the input space so that this modified subset of features would now be unusable by the model *f*. We choose a *zeroing* perturbation function *P*_∅_(*x, R*), which sets to 0 a subset of feature representation of *x* — in our case, a subset of *k* ROIs chosen uniformly at random for each batch *X*_*B*_. For each original data point *x* ∈ *X*_*B*_ and its perturbed counterpart, $\tilde{x}\in {\tilde{X}}_{B}$ we compare their prediction *p* = *f*(*x*) and $\tilde{p}=f\left(\tilde{x}\right)$ and note if they differ. If they do, we add one to the batch score


$$\begin{array}{c} s=\frac{1}{\left|X_{B}\right|}\underset{x\in X_{B}}{\sum }1_{p\neq \tilde{p}} \end{array}$$

In other words, the score *s* is a percentage of predictions in the perturbed batch differing from the predictions in the original batch. We store it in a buffer, for each zeroed ROI *r* ∈ *R* in this batch. The per-batch scores *s* are collected over multiple iterations and batches for each *r* ∈ ROIs, and their average is the final importance score *S*[*r*].

The procedure was run for *N*= 10,000 iterations, with *k* = 12 out of 116 ROIs zeroed in each batch – the choice was arbitrary, but it involves a reasonable trade-off between the coverage of potential multichannel interactions and the computational cost of the procedure (with the number of iterations limited as above, one could only cover all pairwise interactions). A dropout layer in the model architecture enhances parameter randomization, promoting the generalization of the identified ROIs. Still, using a probabilistic scoring method did not significantly affect the results.

### 4.3 Evaluation metrics

We evaluated precision, recall, *F*_1_, and classifier convergence times as suggested in Taha and Hanbury (2015). Our main focus was on *F*_1_ scores and convergence times. Given that the data had varying numbers of samples for each class, we used the weighted micro-averaged *F*_1_ score to account for the class imbalance. The *F*_1_ formula below shows how the final score is calculated, given precision, recall, and *F*_1_ score for each class *i*. This ensures that each class's contribution is proportional to its prevalence in the data set.


$$\begin{array}{c} Precision=\frac{TP}{TP+FP}\;Recall=\frac{TP}{TP+FN} \end{array}$$

$$\begin{array}{c} F1_{i}=\frac{2\times Precision_{i}\times Recall_{i}}{Precision_{i}+Recall_{i}}\;F1=\frac{\sum_{i=1}^{N}\left(W_{i}\times F1_{i}\right)}{\sum_{i=1}^{N}W_{i}} \end{array}$$

where *TP* is the number of true positives, *FP* is the number of false positives, and *FN* is the number of false negatives. The weights *W*_*i*_ correspond to the number of true samples of class *i*.

## 5 Results

### 5.1 Classifier performance comparison

**Figure 4:**
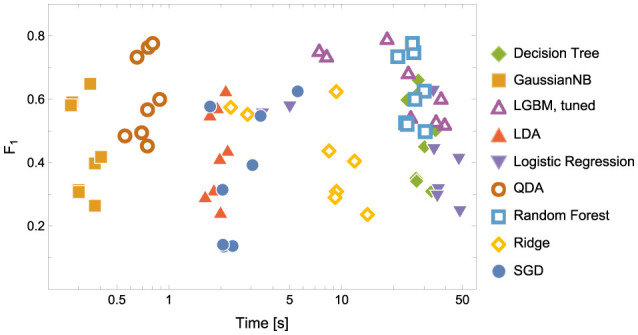
Model Convergence Times and *F*_1_ scores achieved on the test data for discriminators. Notice the logarithmic horizontal time axis. Each point corresponds to the result of a single experiment (i.e., ENC2, ENC3 and so on); cf. the left panel in Figure 5.

**Figure 5:**
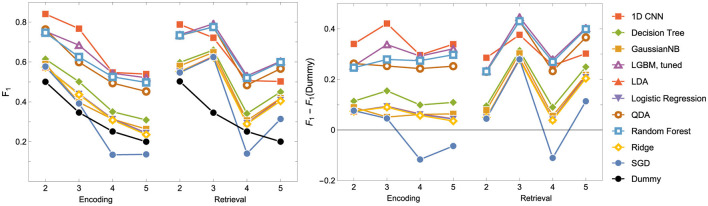
*F*_1_ score for the Encoding and Retrieval experimental phase and different classifiers. For all models other than the CNN, the data used for training were instantaneous brain activations (1 TR × 116 ROI vectors).

As indicated in the Evaluation Metrics subsection, we made final comparisons based on *F*_1_ and convergence time. The combined results can be seen in Figures 4, 5, and Table 5.

**Table 5:** Model evaluation results—*F*_1_ scores achieved on the test data (single time point: 1 TR × 116 ROIs).

**Model**	**Classifier type**	**ENC2**	**ENC3**	**ENC4**	**ENC5**	**RET2**	**RET3**	**RET4**	**RET5**
Dummy classifier	Baseline	0.507	0.347	0.247	0.200	0.504	0.353	0.252	0.204
Ridge classifier	Linear	0.577	0.438	0.306	0.237	0.551	0.621	0.287	0.402
Logistic regression	Linear	0.577	0.442	0.311	0.245	0.551	0.622	0.292	0.406
SGD classifier	Linear	0.535	0.455	0.206	0.084	0.544	0.622	0.142	0.283
Gaussian naive Bayes	Linear	0.583	0.395	0.316	0.264	0.577	0.651	0.299	0.416
Decision tree classifier	Nonlinear	0.615	0.505	0.355	0.318	0.601	0.664	0.342	0.443
Random forest classifier	Nonlinear	0.751	0.634	0.533	0.508	0.734	0.780	**0.532**	0.612
Linear discriminant analysis	Linear	0.577	0.442	0.314	0.247	0.551	0.627	0.292	0.409
Quadratic discriminant analysis	Nonlinear	**0.758**	0.598	0.492	0.456	0.730	0.777	0.487	0.569
LGBM classifier	Nonlinear	0.715	0.631	0.467	0.443	0.697	0.756	0.458	0.545
LGBM classifier tuned	Nonlinear	0.754	**0.684**	**0.543**	**0.521**	**0.737**	**0.792**	0.530	**0.603**

Bold values indicate the best score in a given column.

Based on the results, a few major conclusions can be drawn.

1. In terms of model convergence times and *F*1 scores, the nonlinear classifiers achieved the best performance (see Figure 4). Additionally, Quadratic Discriminant Analysis achieved the highest *F*1 score in the shortest amount of time, at least one order of magnitude less than other nonlinear discriminators.
2. The nonlinear models have shown better accuracy compared to the linear models when examining the experimental data split into encoding and retrieval phases (see Figure 5). The *F*1 measure tends to decrease as the number of considered classes for the encoding phase increases. However, after correcting the results with dummy classifiers, a nearly constant relationship between accuracy and the number of classes is observed. Furthermore, including the resting state in tasks improves the performance of the discriminators, especially during the retrieval phase, where tasks involving the resting state achieve the highest accuracy. This also highlights differences in the data structures recorded in different experimental phases.
3. When looking at the results for the discriminators in Table 5, we found that the best model accuracy came from the tuned LGBM Classifier (nonlinear discriminators). The only exceptions were ENC2 and RET4, where Quadratic Discriminant Analysis and random Forest Classifiers performed slightly better than the tuned LGBM Classifier, respectively.

### 5.2 Classifiers based on neural networks

**Figure 6:**
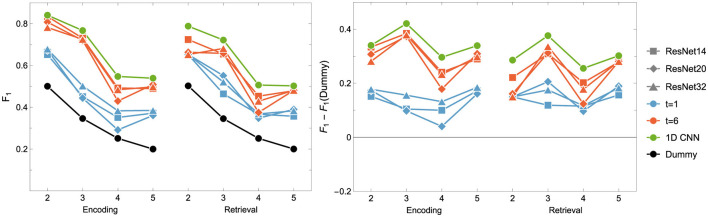
*F*_1_ score for the Encoding and Retrieval experimental phase and different neural networks: ResNet (blue and orange) and (green) CNN. The data used for training were either instantaneous brain activations (*t* = 1; 1 TR × 116 ROIs) or data segments of 6 time points (*t* = 6; 6 TRs × 116 ROIs) for comparison with the other classifiers. CNNs used only *t* = 6 data format.

In our study, we applied CNNs and ResNets with various parameters (e.g., the number of residual blocks). Results on their performance are collected in Figure 6 and Table 6. We hypothesized that models processing temporal-spatial data can yield better results than using single-time observations. Consequently, we involved two approaches as mentioned above, i.e. as an input to ResNet, we used 1-time point data related to the instantaneous view of the brain state and 6-time points corresponding to the dynamics of the brain during the processing of the tasks.

**Table 6:** Neural retworks evaluation results—*F*_1_ scores achieved on the test data.

**Input length**	**Model**	**ENC2**	**ENC3**	**ENC4**	**ENC5**	**RET2**	**RET3**	**RET4**	**RET5**
1 time point	ResNet14	0.652	0.451	0.351	0.372	0.652	0.464	0.367	0.357
	ResNet20	0.675	0.444	0.292	0.361	0.652	0.551	0.348	0.390
	ResNet32	0.678	0.502	0.383	0.385	0.652	0.521	0.368	0.383
6 time points	ResNet14	0.834	0.731	0.492	0.490	0.724	0.655	0.453	0.479
	ResNet20	0.808	0.720	0.430	0.509	0.663	0.658	0.375	0.481
	ResNet32	0.782	0.725	0.484	0.498	0.652	0.682	0.429	0.481
6 time points	1D CNN	**0.841**	**0.767**	**0.547**	**0.539**	**0.788**	**0.722**	**0.506**	**0.502**

The input data used for training were either brain activations during 1 time point (1 TR × 116 ROIs) or data segments of 6 time points (6 TRs × 116 ROIs). Bold values indicate the best score in a given column.

It can be easily drawn that the networks give more accurate results when data in 6-time point segments are used to train the model. Moreover, the higher number of residual blocks within the middle layer does not imply better network performance, which is evident when results for the encoding phase for the 1-time point and the 6-time point are compared. For the former case (1-time point), the best results are obtained for ResNet32, whereas in the latter case, ResNet14 gives better outcomes. Interestingly, the model outperforms the non-neural classifiers only for ENC2 and ENC3 cases. Adding the resting state to the tasks enhances the accuracy only when four classes are considered, i.e. *F*1 for ENC4 (RET4) is lower than ENC5 (RET5). The best 1D CNNs found have performance better than any of the considered ResNets, even though they have a considerably simpler architecture. Interestingly, for the encoding phase, they perform comparably or better than any non-neural classifier, but are comparable or worse for the retrieval phase.

### 5.3 Explainability: ROI importance

**Figure 7:**
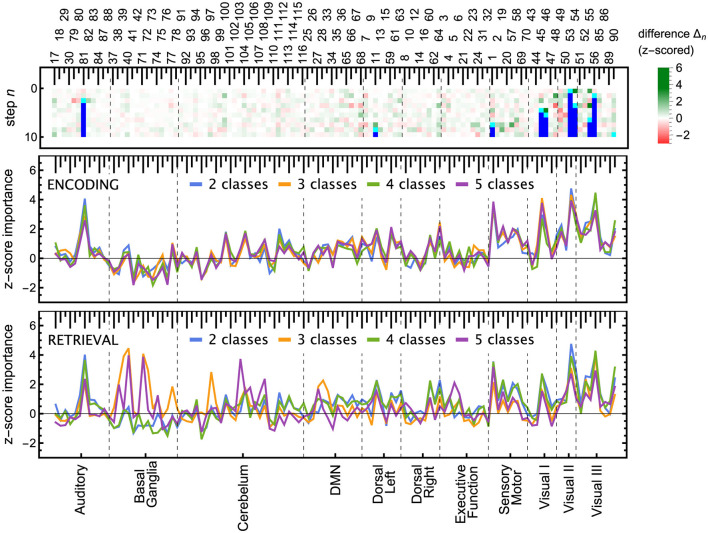
ROI importance scores of the tuned LGBM model, taking into account feature correlations. **(Top)** For the ENC2 experiment, the heatmap shows the change of importance score $\Delta_{n}=s_{R\backslash \left\{r^{*}\right\}}\left(r\right)-s_{R}\left(r\right)$ after iteratively removing the *n*-th most important ROI (only the first ten steps are shown). These changes indicate correlations between features. Cyan (blue) pixels—regions removed in a given step *n* (in a previous step); green (red) pixels—regions whose importance increased (decreased) due to the removal. See Figure 11 for the other experiments. Note, for instance, the increased importance of the right inferior occipital (54) and left fusiform gyrus (55) after removing the left inferior occipital gyrus (53). Cf. results in the bottom panels and the unmodified split importance in Supplementary Figure 1. **(Middle and bottom)** The panels show the median over all iterations until the removal of a given ROI of the *z*-scored importance *S*(*r*). These scores account for the increases and drops visible in the top panel due to ROI correlations. ROIs are ordered according to the resting-state networks given at the bottom. The ROI numbering of AAL atlas is given at the top; see Supplementary material for ROI names.

The ROI pruning procedure, Algorithm 1, allowed us to see whether, after removing a highly significant feature r^*^, some other remaining ROIs considerably changed their scores. Such changes are visualized in the upper panel of Figure 7 and in Figure 11, where in each row of the heatmap green (red) pixels indicate the regions whose importance increased (decreased) due to the removal of a single ROI (cyan tips of blue vertical lines). These changes can be interpreted as the model compensating for the information loss due to the r^*^ removal by using similar information obtained from the several remaining ROIs, whose importance increased. We observed those remaining features improving their importance scores, with just a small overall *F*_1_ performance decrease. The importance of the other remaining ROIs would decrease if the information they carried was only useful in combination with the one that had been removed.

**Figure 8:**
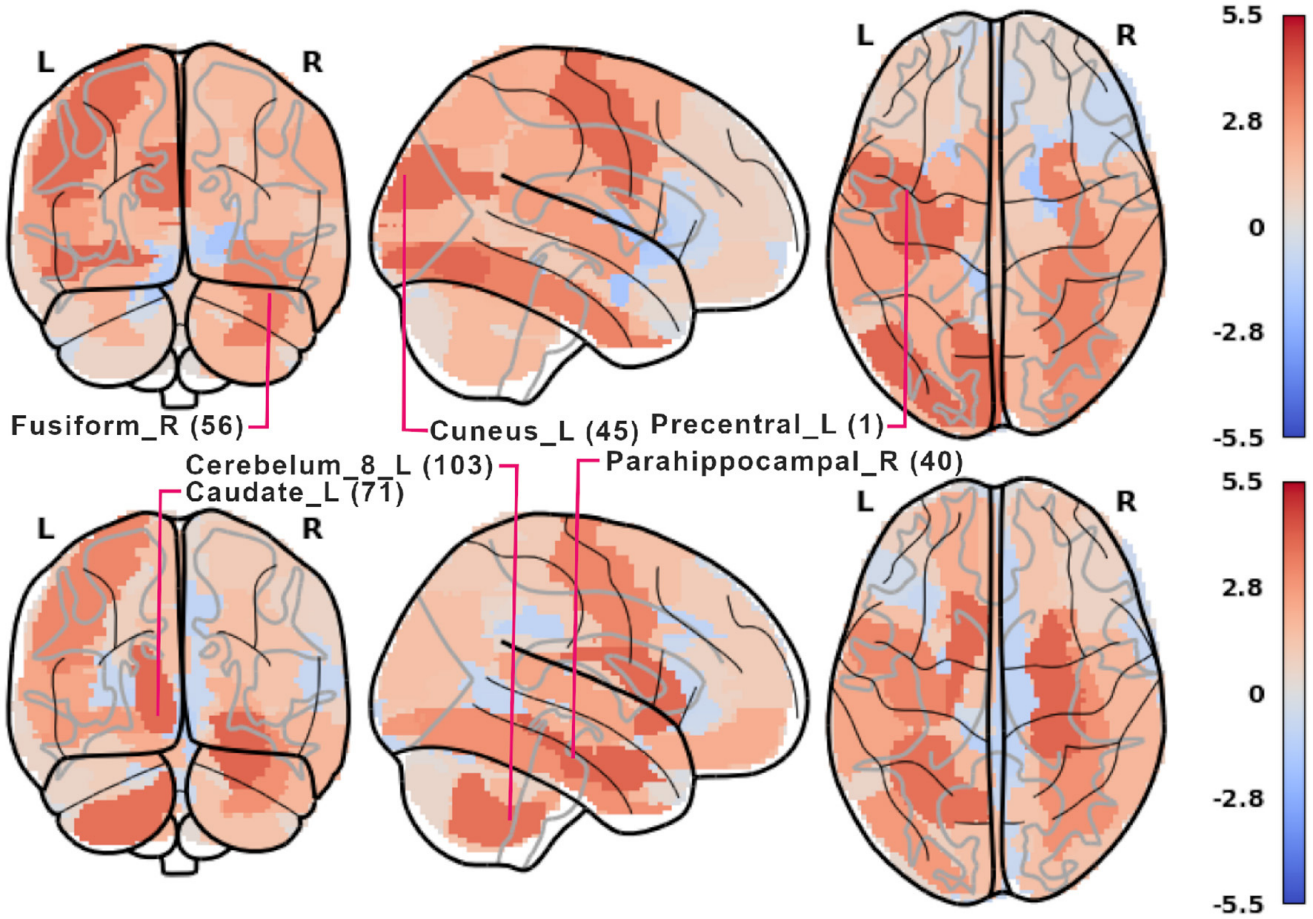
Plot of *z*-scored LGBM importance from Figure 7 in 5-class encoding **(top)** and retrieval **(bottom)**. For each phase, some of the most important AAL ROIs are labeled. 2-, 3-, and 4-class results are shown in Supplementary material. Rendered with nilearn (Abraham et al., 2014).

Notable examples of correlated clusters of highly important ROIs are the ones responsible for visuals: 53, 54, and 55 (left inferior occipital gyrus, right inferior occipital, and left fusiform gyrus) or 48 and 56 (right lingual gyrus and right fusiform gyrus) appearing in encoding and retrieval experiments without resting state; see Figure 7. Similarly, certain basal ganglia (39, 40, 71, 72) and some cerebellar areas (97, 103, 105, 108) had correlated scores and were important for the classifier's decisions in encoding experiments against the resting state. The full list of ROI names can be found in Supplementary material. Figure 8, together with Supplementary Figures 2–4, offer a visualization of where the most important brain regions are located.

The ROI importance scores (not corrected for possible correlations) of the tuned LGBM model are shown in Supplementary Figure 1, together with all the 100 optimisation trials in Supplementary Figures 5, 6. Comparing these scores with the ones obtained from pruning, one can notice where the procedure was indeed beneficial. For instance, before pruning (Supplementary Figure 1) the right inferior occipital gyrus (54) had low importance, while the left inferior occipital gyrus (53) was highly important, but after pruning (Figure 7) both areas are almost equally highly important. Similarly, before pruning the right middle occipital gyrus (52) was considerably more important than the left middle occipital gyrus (51), but after pruning in most of the experiments they both have medium importance.

**Figure 9:**
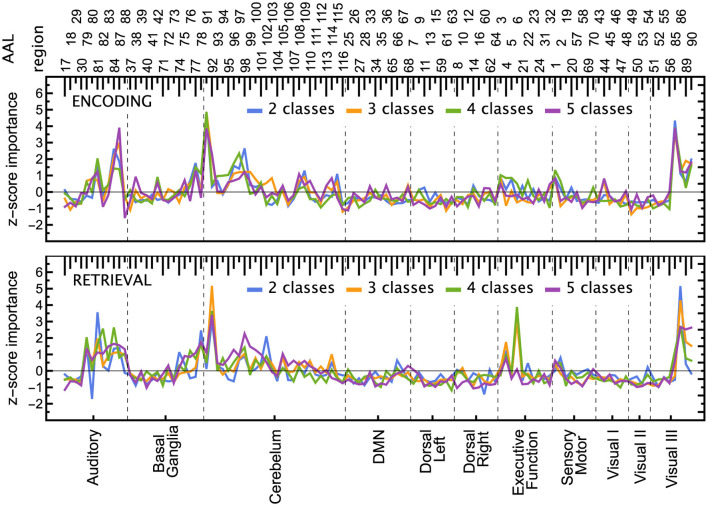
ROI importance scores for the ResNet. The scores were calculated as described in Algorithm 2 with additional *z*-scoring for comparison with Figure 7.

**Figure 10:**
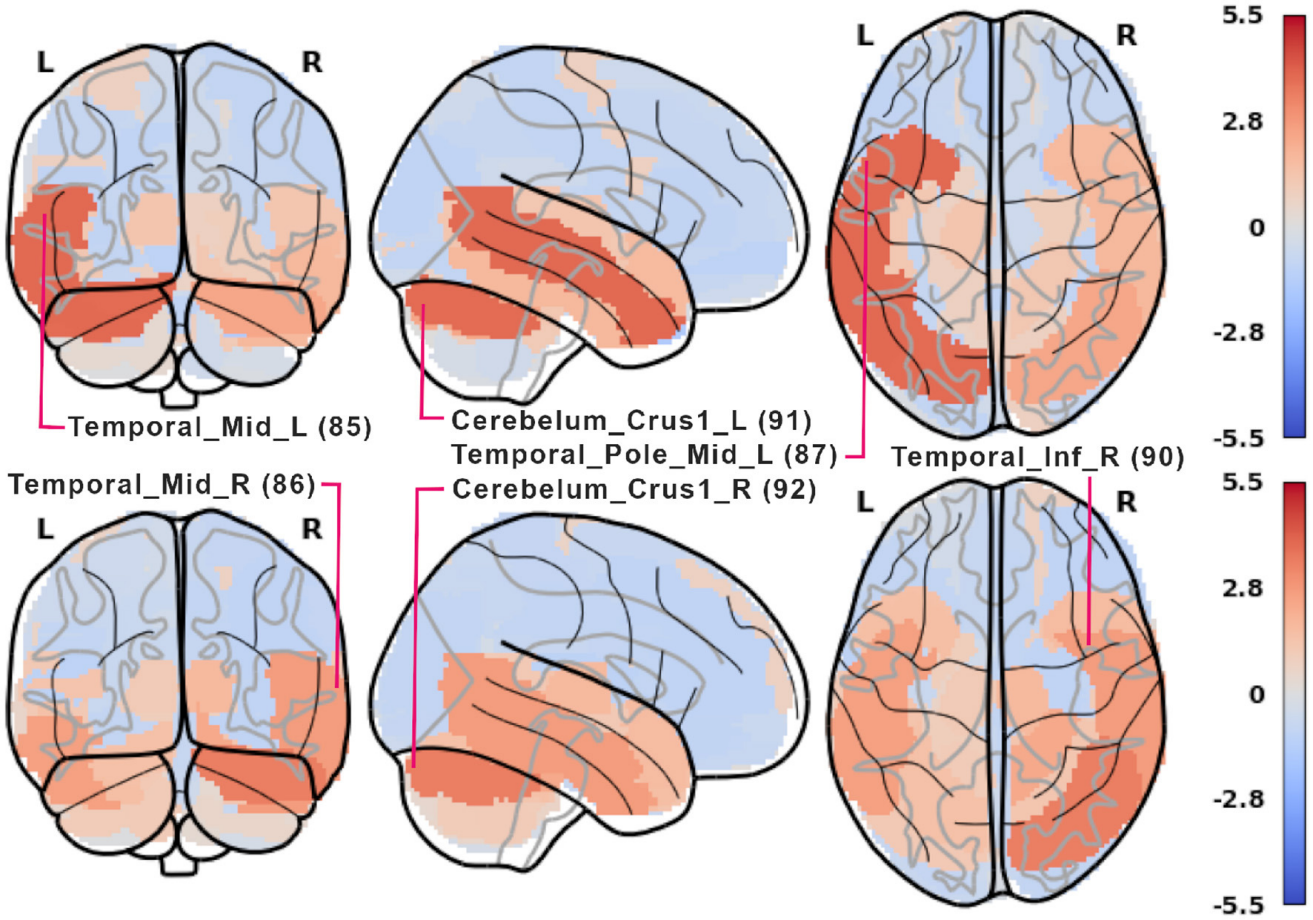
Plot of *z*-scored ResNet importance from Figure 9 in 5-class encoding **(top)** and retrieval **(bottom)**. For each phase, the three most important AAL ROIs are labeled. 2-, 3-, and 4-class results are shown in Supplementary material. Rendered with nilearn (Abraham et al., 2014).

The respective results obtained from ResNets and Algorithm 2 are shown in Figures 9, 10 and in Supplementary Figures 9–13. Fewer ROIs than in the previous method reach high scores, notably: 85, 86 visual processing regions, 4 and 6 executive regions, 91 and 92 in the cerebellum, and several auditory ROIs (e.g., 81, 84, 87).

Surprisingly, there is no significant correlation between respective ResNet and LGBM importance scores, even though their classification performance is comparable. For 2- and 4-class tasks the encoding and retrieval scores are less correlated for ResNet (correlation coefficient 0.27 and 0.55, respectively) than LGBM (0.94 in both cases), but are similarly correlated for 3- and 5-class tasks (0.48 and 0.57 versus 0.38 and 0.50). The correlation between 2- and 4-class importance scores (0.88 for ENC and 0.76 for RET) and 3- and 5-class (0.89, 0.80) are comparable with LGBM (0.91, 0.92, 0.95, 0.68, respectively).

## 6 Discussion

From the neurocognitive perspective, the discriminant analysis allows us to reveal the dynamic process of working memory. As visualized in Figure 7, the first 10 steps of pruning showed important ROIs mainly in auditory, sensory-motor, and visual networks in both encoding and retrieval phases. Superior temporal gyrus and temporal pole (ROIs 81, 82, 83) from the auditory network are responsible for the encoding of speech sounds (Yi et al., 2019), speech representation (Chang et al., 2010), or visual cognition (Herlin et al., 2021). In the 2-class task, when the distinction between visuospatial and verbal tasks was made, these regions seem to play a significant role (see brain maps in Supplementary Figure 2). In the visual networks, the important regions are inferior occipital gyri (ROIs 53, 54; see brain maps in Figure 8), which are involved in spatial feature processing (de Haas et al., 2021) as well as in insightful problem solving (Qiu et al., 2010). The results are in line with the sensory reactivation theory, according to which the retrieval phase involves the reinstatement of a process that appeared in the encoding phase (Abe, 2012).

**Figure 11:**
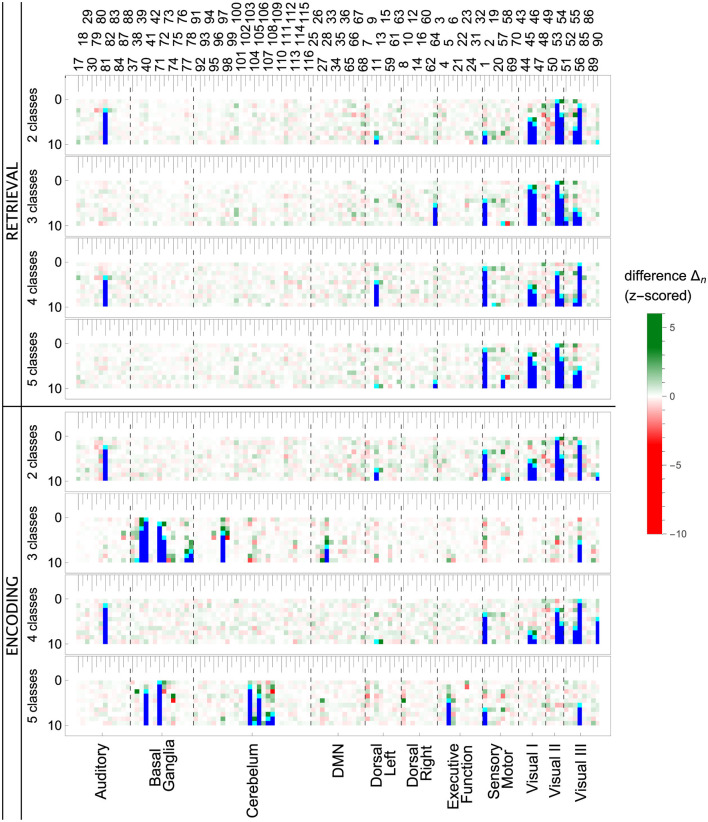
Influence of feature pruning on importance scores of the LGBM model. Each colored row in a matrix represents standardized differences in importance scores after dropping one feature. The feature dropped in a given step is cyan, and all features dropped before are blue. ROIs whose importance increased (decreased) in a given step are green (red). Full matrices with 115 pruning steps are shown in the Supplementary material.

When we look at the influence of feature pruning on importance scores (see Figure 11), it can be easily noticed that all experiments in the encoding phase are very similar, but differences are seen in the retrieval stage. Regarding the results from experiments during encoding, the visual regions (like ROIs 46, 47, 53, 54) gain higher importance, which suggests the differences in the involvement of visual networks related to memorizing different visual stimuli. In the retrieval phase, we also observed the significance of visual brain areas, which could be interpreted using the sensory reactivation hypothesis. However, our results showed mainly the importance of regions in the Visual III network, namely the fusiform gyri and inferior temporal gyri (ITG). The fusiform gyri are engaged in high-level information processing such as object recognition, visual language perception, and visual attention (Caspers et al., 2014). They are defined as critical structures for visual categorization (Grill-Spector and Weiner, 2014), whereas the inferior temporal gyri are also involved in visual object recognition, as well as in phonological and lexical processing, and decision-making. ITG is assumed to be a key region in the visual association area with connections from visual regions and high-order areas including the prefrontal cortex (Lin et al., 2020). Our analysis revealed the importance of the superior frontal gyrus, a part of the medial prefrontal cortex, which is engaged in higher levels of working memory processing like monitoring and manipulation and in spatial feature processing (Boisgueheneuc et al., 2006). The study by Hu et al. (2016) showed also the role of the superior frontal gyrus in executive control, more specifically in efficient response inhibition. The results of a recent study using a combination of electrocorticography and direct cortical stimulation suggested that this structure might be a key node coordinating working memory (Alagapan et al., 2019). Our results showing the significance of inferior temporal and superior frontal gyri in retrieval phase seem to confirm the evidence that content representations during encoding and retrieval in WM, differ and engage distinct brain regions. Specifically, the importance of the superior frontal gyri indicates the conversion of content representations from visual to frontoparietal regions (Favila et al., 2020; Fafrowicz et al., 2023). The applying machine learning algorithms allowed us to achieve results that go beyond the sensory reactivation hypothesis. They seem to be in line with the recent evidence showing differences in neural activity patterns of encoding and retrieval processes, revealing change in the neural localization of content representations (Favila et al., 2020; Fafrowicz et al., 2023).

In RET3 and RET5 (the experiments including REST), the regions in basal ganglia and cerebellum networks scored the highest importance. The basal ganglia were shown to be involved in the control of attention to problem features, as well as the transport of information between higher visual regions and the prefrontal cortex. There is also evidence of the basal ganglia-cerebellum-prefrontal cortex network, whose activation is associated with working memory and executive functions (Bostan and Strick, 2018). Current computational models shed new light on working memory as a process that strikes a trade-off between stability and flexibility (the core feature of executive control) controlled by the basal ganglia and cerebral cortex (Trutti et al., 2021). It is well known that dopamine is a key transmitter in the working memory process (Ott and Nieder, 2019). The recent study revealed the role of a balance between prefrontal and striatal dopamine secretion and dynamic dopamine-dependent adjustment for adaptive cognition. The results showed the influence of basal ganglia on cognitive control modulation in a way that striatal dopamine controls flexible gating of actions with increasing activity in the direct “go” pathway and decreasing activity of the indirect “no-go” pathway of this structure (Cools, 2019). Regarding the cerebellum, recent studies have shown that this structure is involved in a much higher number of cognitive functions than was assumed (Stoodley, 2011; Schmahmann, 2018). The basal ganglia and cerebellum regions seem to be key regions in the distinction between cognitive and non-cognitive (REST) tasks, which to our knowledge has been confirmed for the first time in the present neural networks analysis.

Regarding the ResNet model results (see Figure 9), high ROI importance was attributed to cerebellum regions and higher-order visual processing areas (Visual III network). Notably, during the retrieval phase, ResNets place importance on the executive network, which is not observed in the LGBM outcomes (shown in Figure 7). This suggests that ResNet may be more effective at identifying structures involved in a task, even though LGBM's importance scores are more consistent. Additionally, when examining the three most important ROIs in the ResNet model (see Figure 10), we observe differences in hemispheric locations between the encoding and retrieval phases. These differences seem to support the newly established model of Activity Silent Working Memory, highlighting the involvement of episodic memory in working memory tasks related to context representation (for the review, see Beukers et al., 2021).

The importance scores of the LGBM model, on the other hand, are remarkably similar to the results obtained from measuring temporal correlations in the same data (Ochab et al., 2022). In particular, the differences in Hurst exponents between the perceptual (GLO, LOC) and verbal similarity (PHO, SEM) tasks pointed to the same regions in Sensory Motor, Visual I and Visual II networks, and partly in Dorsal networks for the encoding phase; a similar pattern in Sensory Motor, Visual I and Visual II networks appeared also in the retrieval phase. There are no such striking similarities with the ResNet results. This observation is intriguing, since the LGBM had no access to the temporal information (classification based on single time points), whereas the Hurst exponents quantify the temporal correlation structure of time series. And vice versa, the Hurst exponents were computed separately for each ROI, whereas LGBM results depend solely on the equal-time cross-correlations between ROIs. Such interconnection between autocorrelation and cross-correlation in fMRI data has been previously observed in Ochab et al. (2019).

Our results based on the new methods of data analysis confirmed the dynamic-processing model of working memory. This process is much more stable in the encoding phase than in the retrieval phase. This stability related to the capacity-limited WM process can be seen in the similarity of the encoding phase, where the differences are revealed only in the visual processing regions regarding specific characteristics of visual stimuli. The retrieval of information from memory is more flexible (changing over time) and is context-dependent. We also observed the change in the neural localization from visual to frontoparietal regions, which is not in line with the sensory reactivation hypothesis. To conclude, we believe that the application of some of the most commonly used machine learning algorithms and neural networks to investigate the encoding and retrieval phases is very promising for future research.

## Data Availability

The original contributions presented in the study are included in the article/Supplementary material, further inquiries can be directed to the corresponding author.
