# Supplementary material for: Classification of ROI-based fMRI data in short-term memory tasks using discriminant analysis and neural networks
Source: Front Neuroinform. 2024 Dec 20;18:1480366. doi: 10.3389/fninf.2024.1480366 (PMC11695337; doi:10.3389/fninf.2024.1480366)
Supplement: Supplementary file 1 [file Data_Sheet_1.pdf]

Supplementary information to *Classification of ROI-based fMRI data in short-term memory tasks using discriminant analysis and neural networks*

Magdalena Fąfrowicz[4], Marcin Tutajewski[1], Igor Sieradzki[3], Jeremi K. Ochab[1,2,*], Anna Ceglarek-Sroka[4], Koryna Lewandowska[4], Tadeusz Marek[5], Barbara Sikora-Wachowicz[4], Igor T. Podolak[3], and Paweł Oświęcimka[6,1,2,]**

[1]Institute of Theoretical Physics, Jagiellonian University, 30-348 Kraków, Poland
[2]Mark Kac Complex Systems Research Centre, Jagiellonian University, 30-348 Kraków, Poland
[3]Group of Machine Learning Methods GMUM, Faculty of Mathematics and Computer Science, Jagiellonian University, 30-348 Kraków, Poland
[4]Department of Cognitive Neuroscience and Neuroergonomics, Jagiellonian University, 30-348 Kraków, Poland
[5]Faculty of Psychology, SWPS University, 40-326 Katowice, Poland
[6]Complex Systems Theory Department, Institute of Nuclear Physics, Polish Academy of Sciences, 31-342 Kraków, Poland
[*]jeremi.ochab@uj.edu.pl
[**]pawel.oswiecimka@ifj.edu.pl

ABSTRACT

Understanding the brain's functioning relies on identifying spatiotemporal patterns in brain activity. In recent years, machine learning methods have been widely used to detect connections between regions of interest (ROIs) involved in cognitive functions, as measured by the fMRI technique. However, it's essential to match the type of learning method to the problem type, and extracting the information about the most important ROI connections is challenging. In this contribution, we used machine learning techniques to classify tasks in a working memory experiment and identify the brain areas involved in processing information. We utilized classical discriminators and residual neural networks to differentiate between brain responses to distinct types of stimuli (visuospatial and verbal) and different phases of the experiment (information encoding and retrieval). The best performance was achieved by the LGBM classifier with 1-time step input data and the residual neural network for 6-time step data segments during the encoding and retrieval phases. Additionally, we developed an algorithm that took into account feature correlations to estimate the most important brain regions for the model's accuracy. Our findings suggest that from the perspective of considered models, brain signals related to the resting state have a similar degree of complexity to those related to the encoding phase, which does not improve the model's accuracy. However, during the retrieval phase, the signals were easily distinguished from the resting state, indicating their different structure. The study identified brain regions that are crucial for processing information in working memory, as well as the differences in the dynamics of encoding and retrieval processes. Furthermore, our findings indicate spatiotemporal distinctions related to these processes. The analysis confirmed also the important role of the basal ganglia in processing information during the retrieval phase. The presented results reveal the benefits of applying machine learning algorithms to investigate working memory dynamics.

This PDF file includes:

AAL list of ROI names and numbering.

Supplementary Figures S1 to S14 .

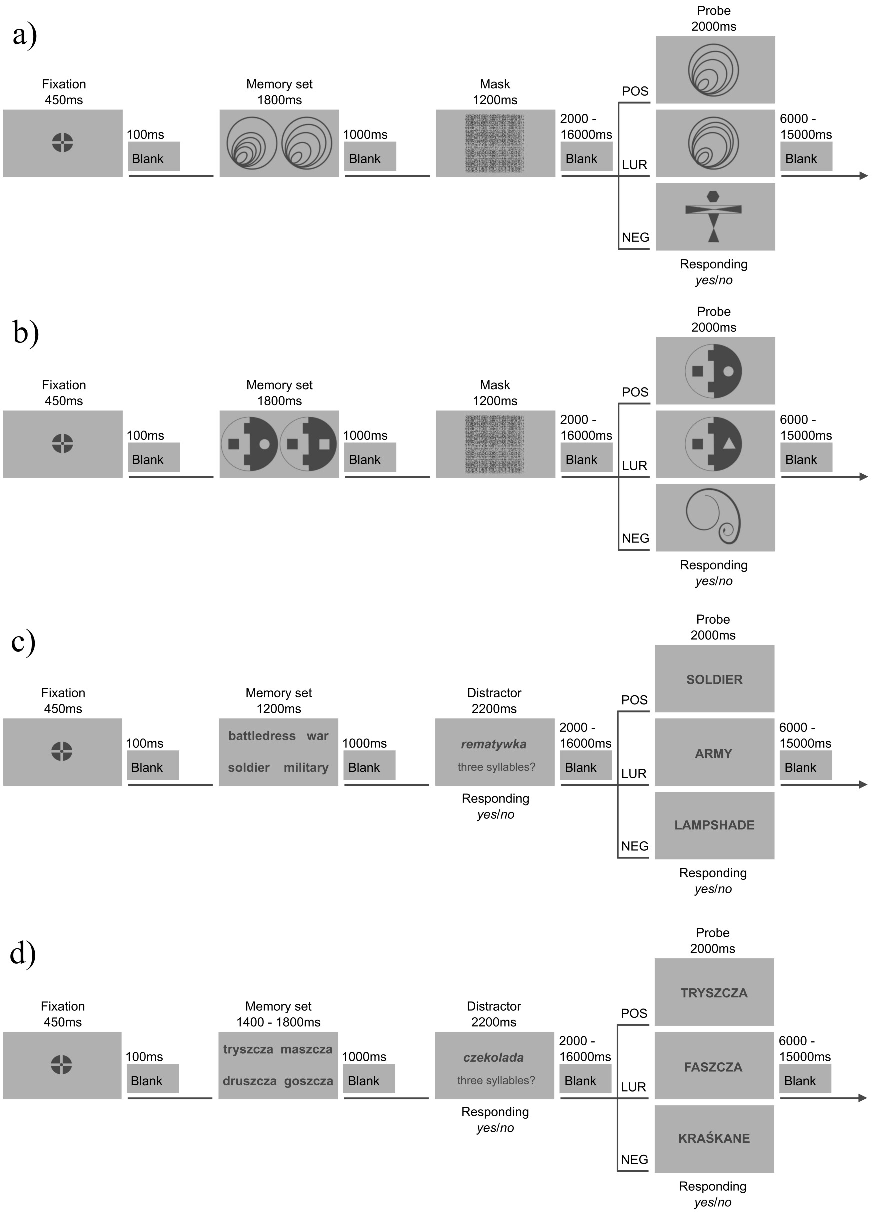

Supp. Figure 1. Experimental tasks: a) global information processing task, GLO; b) local information processing task, LOC; c) semantic task, SEM; d) phonological task, PHO. Visuospatial stimuli were presented in dark gray (RGB 72, 72, 72) on a light gray (RGB 176, 176, 176) background, made with Inkscape [1]. The masks were generated with MATLAB [2]. Verbal stimuli were presented in Calibri 22-point font.

[1]Inkscape. Version 0.92.3. 2018. Inkscape Project. URL:https://inkscape.org/release/inkscape-0.92.3/
[2]MATLAB. Version 9.1.0 (R2016b). 2016. Natick, Massachusetts: The MathWorks Inc.

Index	ROI name	Index	ROI name	Index	ROI name
1	Precentral_L	41	Amygdala_L	81	Temporal_Sup_L
2	Precentral_R	42	Amygdala_R	82	Temporal_Sup_R
3	Frontal_Sup_L	43	Calcarine_L	83	Temporal_Pole_Sup_L
4	Frontal_Sup_R	44	Calcarine_R	84	Temporal_Pole_Sup_R
5	Frontal_Sup_Orb_L	45	Cuneus_L	85	Temporal_Mid_L
6	Frontal_Sup_Orb_R	46	Cuneus_R	86	Temporal_Mid_R
7	Frontal_Mid_L	47	Lingual_L	87	Temporal_Pole_Mid_L
8	Frontal_Mid_R	48	Lingual_R	88	Temporal_Pole_Mid_R
9	Frontal_Mid_Orb_L	49	Occipital_Sup_L	89	Temporal_Inf_L
10	Frontal_Mid_Orb_R	50	Occipital_Sup_R	90	Temporal_Inf_R
11	Frontal_Inf_Oper_L	51	Occipital_Mid_L	91	Cerebelum_Crus1_L
12	Frontal_Inf_Oper_R	52	Occipital_Mid_R	92	Cerebelum_Crus1_R
13	Frontal_Inf_Tri_L	53	Occipital_Inf_L	93	Cerebelum_Crus2_L
14	Frontal_Inf_Tri_R	54	Occipital_Inf_R	94	Cerebelum_Crus2_R
15	Frontal_Inf_Orb_L	55	Fusiform_L	95	Cerebelum_3_L
16	Frontal_Inf_Orb_R	56	Fusiform_R	96	Cerebelum_3_R
17	Rolandic_Oper_L	57	Postcentral_L	97	Cerebelum_4_5_L
18	Rolandic_Oper_R	58	Postcentral_R	98	Cerebelum_4_5_R
19	Supp_Motor_Area_L	59	Parietal_Sup_L	99	Cerebelum_6_L
20	Supp_Motor_Area_R	60	Parietal_Sup_R	100	Cerebelum_6_R
21	Olfactory_L	61	Parietal_Inf_L	101	Cerebelum_7b_L
22	Olfactory_R	62	Parietal_Inf_R	102	Cerebelum_7b_R
23	Frontal_Sup_Medial_L	63	SupraMarginal_L	103	Cerebelum_8_L
24	Frontal_Sup_Medial_R	64	SupraMarginal_R	104	Cerebelum_8_R
25	Frontal_Med_Orb_L	65	Angular_L	105	Cerebelum_9_L
26	Frontal_Med_Orb_R	66	Angular_R	106	Cerebelum_9_R
27	Rectus_L	67	Precuneus_L	107	Cerebelum_10_L
28	Rectus_R	68	Precuneus_R	108	Cerebelum_10_R
29	Insula_L	69	Paracentral_Lobule_L	109	Vermis_1_2
30	Insula_R	70	Paracentral_Lobule_R	110	Vermis_3
31	Cingulum_Ant_L	71	Caudate_L	111	Vermis_4_5
32	Cingulum_Ant_R	72	Caudate_R	112	Vermis_6
33	Cingulum_Mid_L	73	Putamen_L	113	Vermis_7
34	Cingulum_Mid_R	74	Putamen_R	114	Vermis_8
35	Cingulum_Post_L	75	Pallidum_L	115	Vermis_9
36	Cingulum_Post_R	76	Pallidum_R	116	Vermis_10
37	Hippocampus_L	77	Thalamus_L		
38	Hippocampus_R	78	Thalamus_R		
39	ParaHippocampal_L	79	Heschl_L		
40	ParaHippocampal_R	80	Heschl_R		

Table 1. The AAL numbering and ROI short names used.

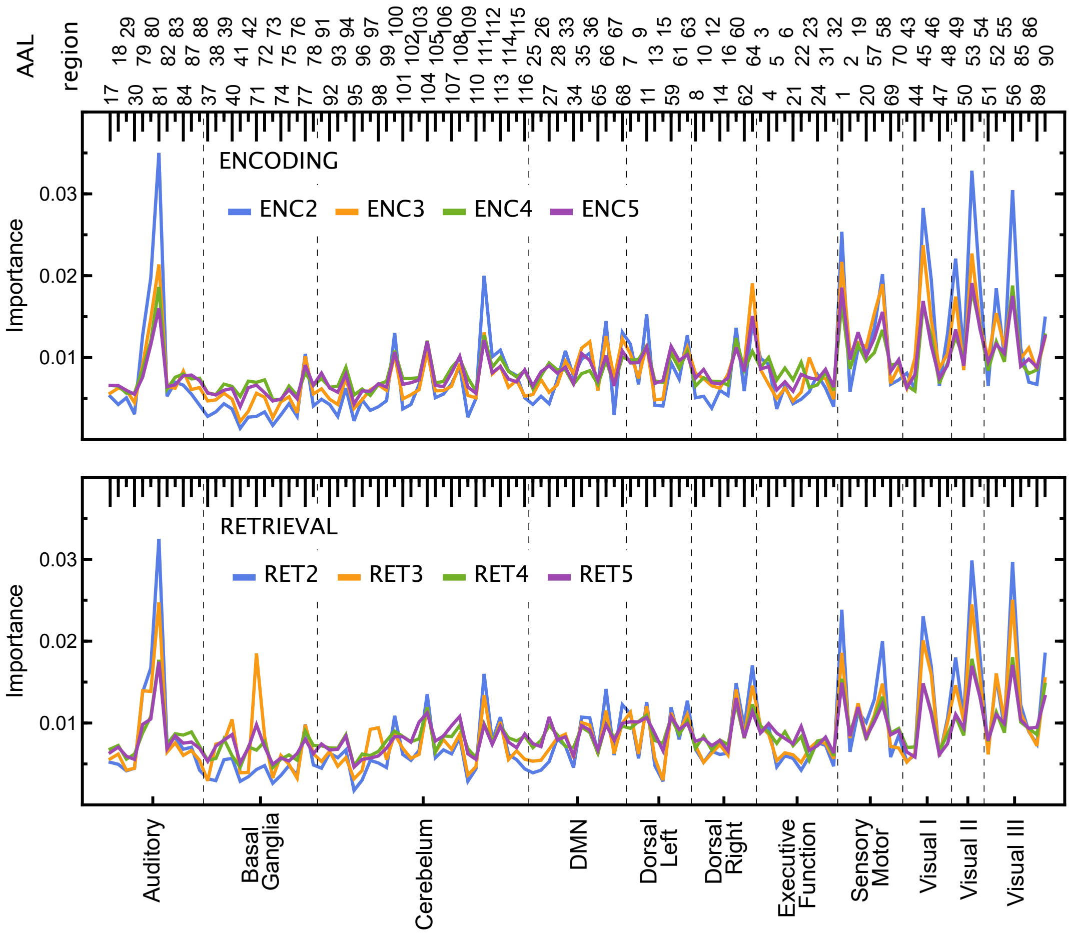

Supp. Figure 2. Split importance scores of the tuned LGBM model for each ROI ordered according to the resting-state networks. The ROI numbering of the AAL atlas is given at the top. No ROI pruning (Alg. 1 in the paper) was performed, so the scores do not explicitly take into account possible feature correlations as in Fig. 6 (in the paper).

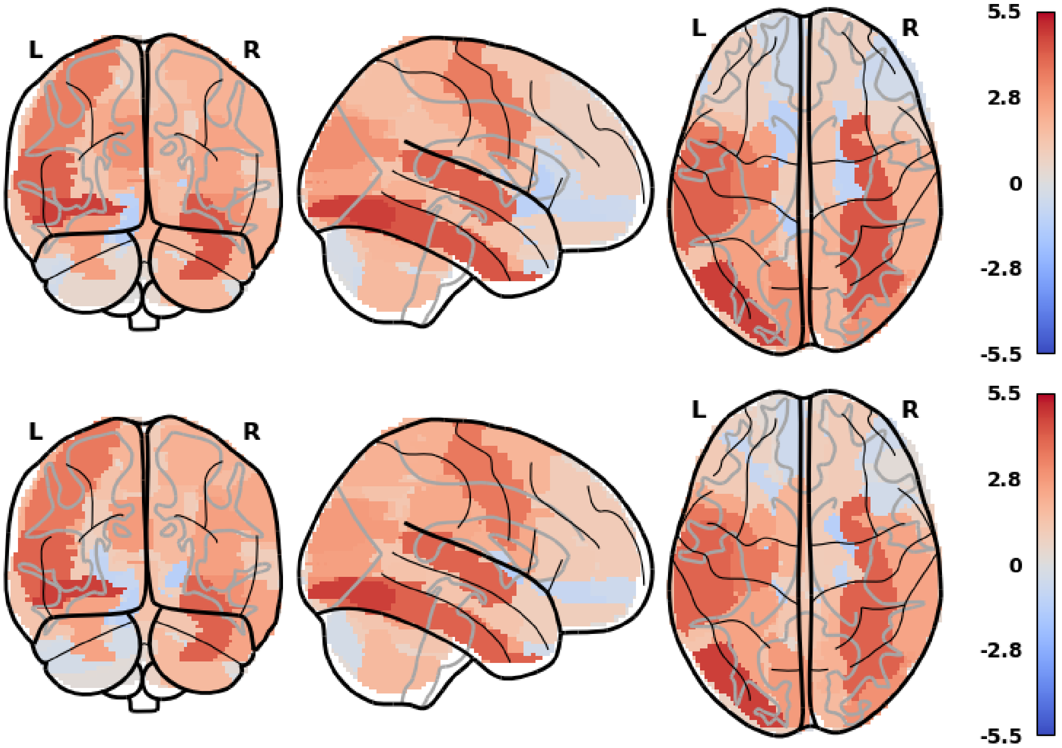

Supp. Figure 3. LGBM importance from Fig. 6 (in the paper) in 2-class encoding (top) and retrieval (bottom). The colour bar shows the z-score of importance.

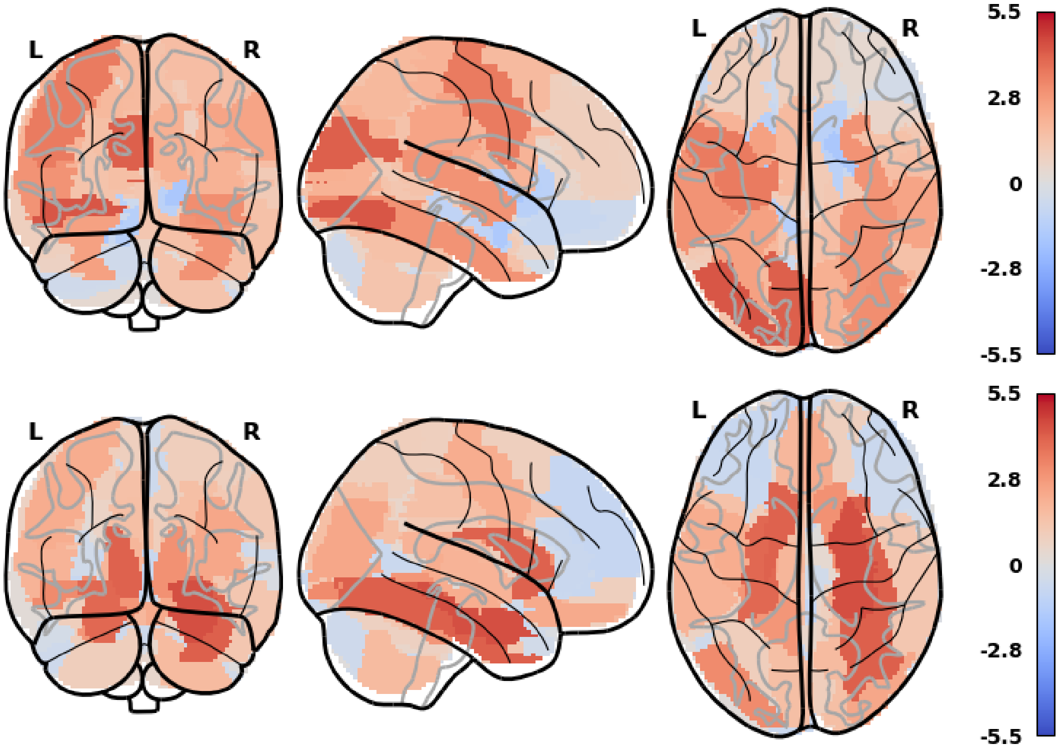

Supp. Figure 4. LGBM importance from Fig. 6 (in the paper) in 3-class encoding (top) and retrieval (bottom). The colour bar shows the z-score of importance.

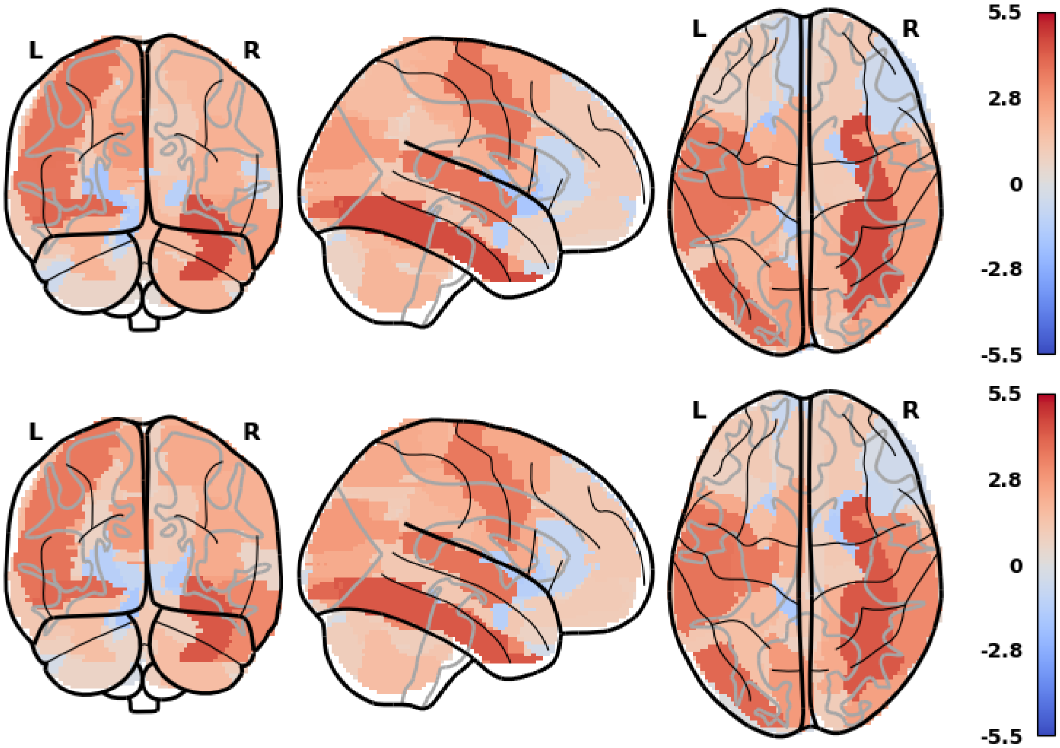

Supp. Figure 5. LGBM importance from Fig. 6 (in the paper) in 4-class encoding (top) and retrieval (bottom). The colour bar shows the z-score of importance.

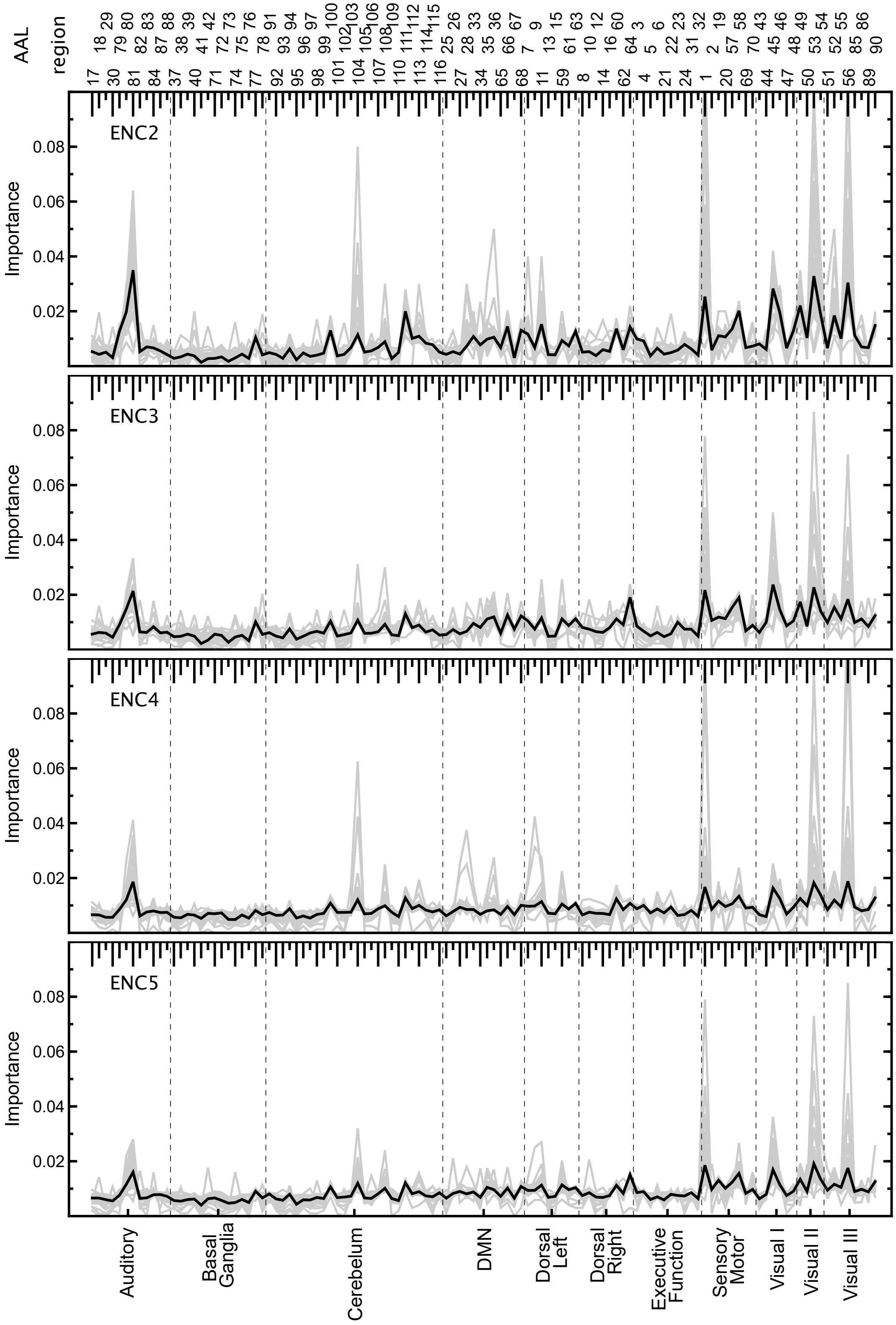

Supp. Figure 6. Split importance scores of the tuned LGBM model for encoding phase. The grey lines are 100 hyperparameter-tuned model versions; the black line is the best version found (shown in Supp. Fig. 2).

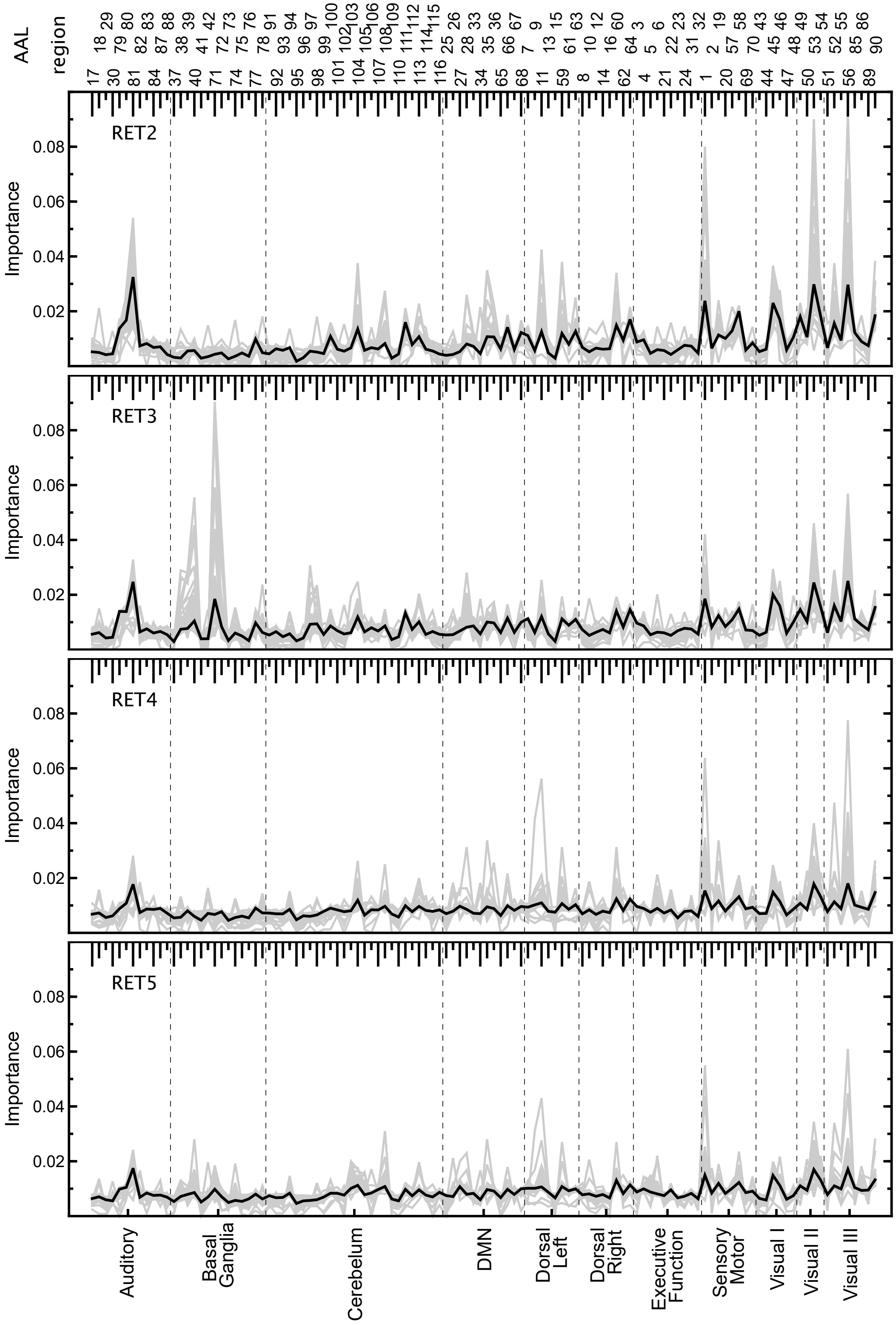

Supp. Figure 7. Split importance scores of the tuned LGBM model for retrieval phase. The grey lines are 100 hyperparameter-tuned model versions; the black line is the best version found (shown in Supp. Fig. 2).

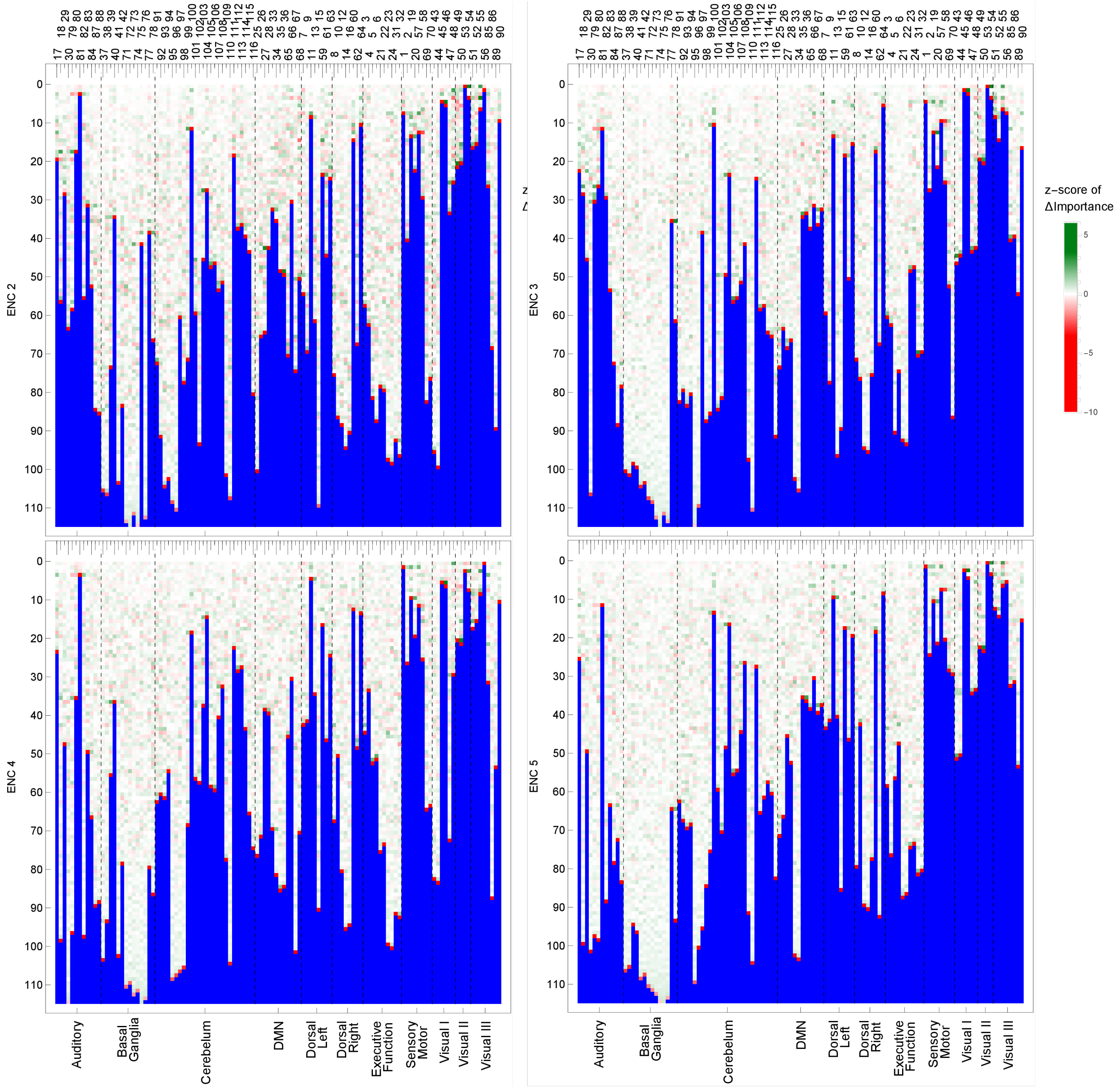

Supp. Figure 8. Influence of feature pruning on importance scores of the LGBM model in encoding. Each coloured row in a matrix represents standardised differences in importance scores, $\Delta_n = s_{R\setminus\{r^*\}}(r) - s_R(r)$, after dropping one feature r^*. The feature dropped in a given step is intensely red, and all features dropped before are blue (the most important features correspond to the highest blue columns). ROIs whose importance increased (decreased) in a given step are green (red).

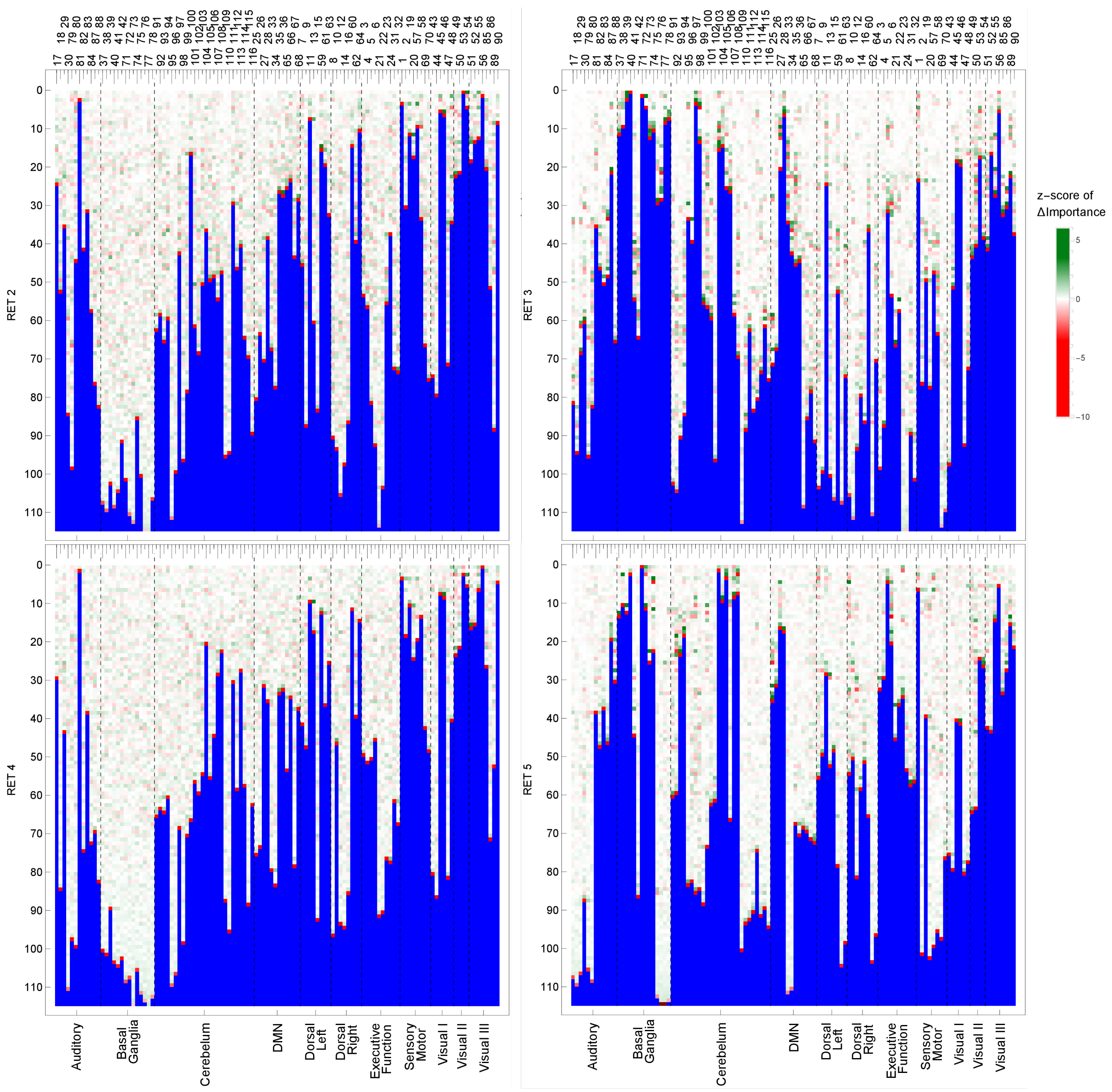

Supp. Figure 9. Influence of feature pruning on importance scores of the LGBM model in retrieval. Each coloured row in a matrix represents standardised differences in importance scores, $\Delta_n = s_{R\setminus\{r^*\}}(r) - s_R(r)$, after dropping one feature r^*. The feature dropped in a given step is intensely red, and all features dropped before are blue (the most important features correspond to the highest blue columns). ROIs whose importance increased (decreased) in a given step are green (red).

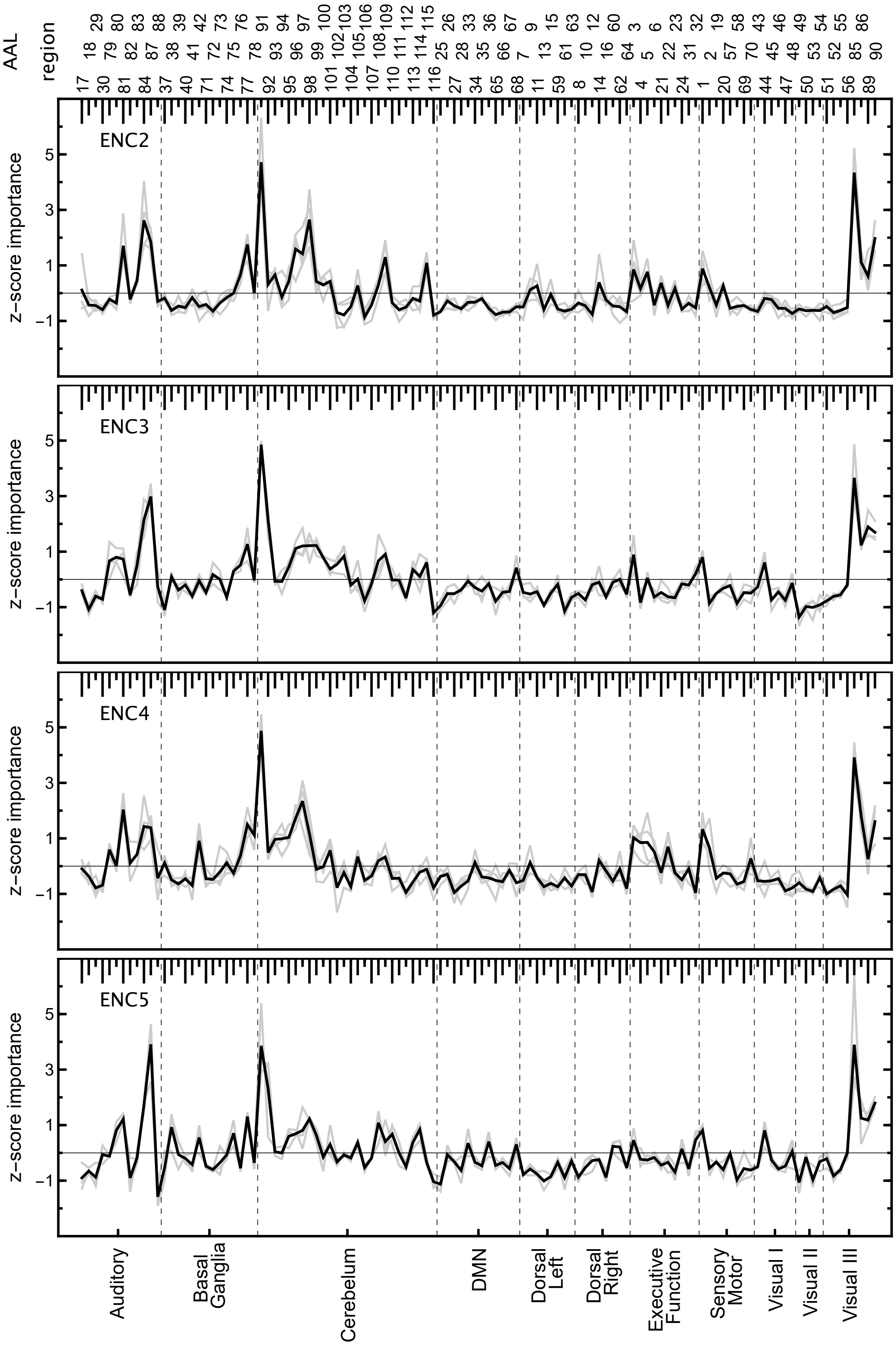

Supp. Figure 10. Importance scores of ResNets for encoding phase. The grey lines are 4 independent realisations; the black line is their mean (shown in Fig. 8 in the paper).

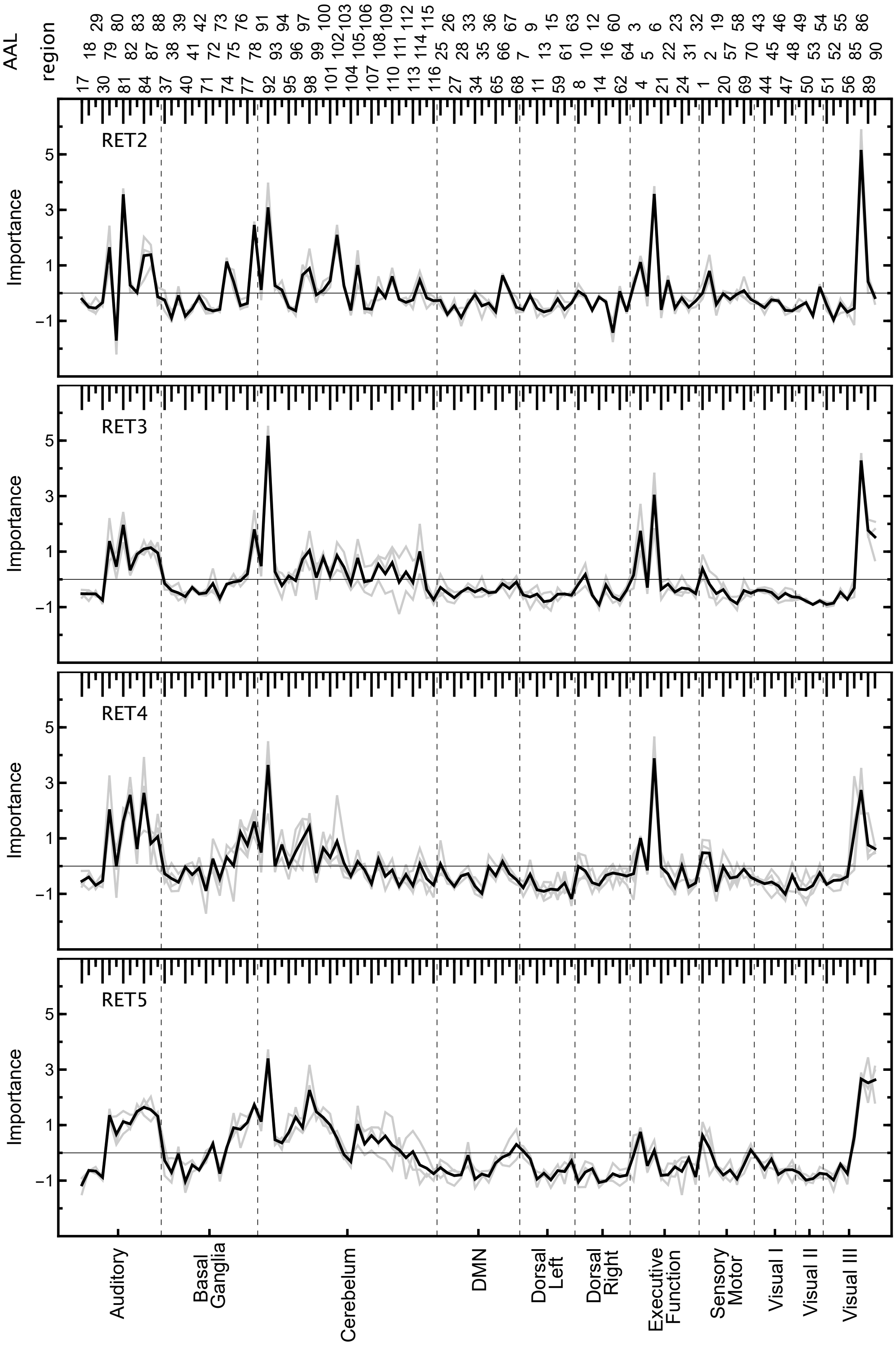

Supp. Figure 11. Importance scores of ResNets for encoding phase. The grey lines are 4 independent realisations; the black line is their mean (shown in Fig. 8 in the paper).

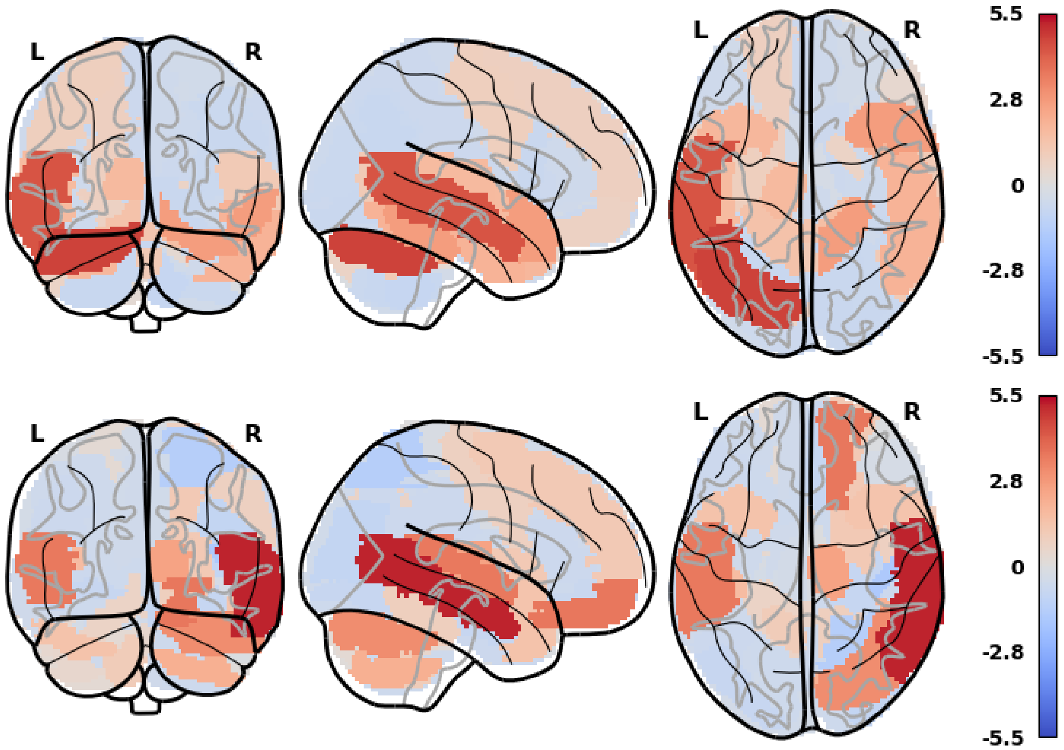

Supp. Figure 12. Z-scored ResNet importance from Fig. 8 (in the paper) in 2-class encoding (top) and retrieval (bottom). The colour bar shows the z-score of importance.

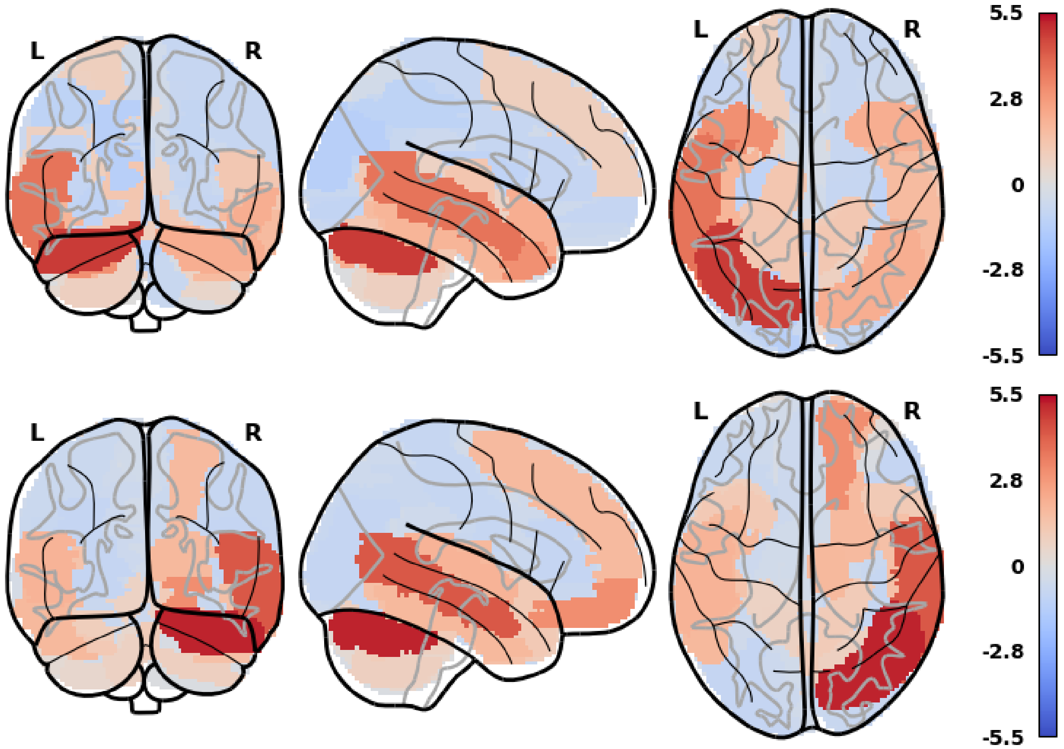

Supp. Figure 13. Z-scored ResNet importance from Fig. 8 (in the paper) in 3-class encoding (top) and retrieval (bottom). The colour bar shows the z-score of importance.

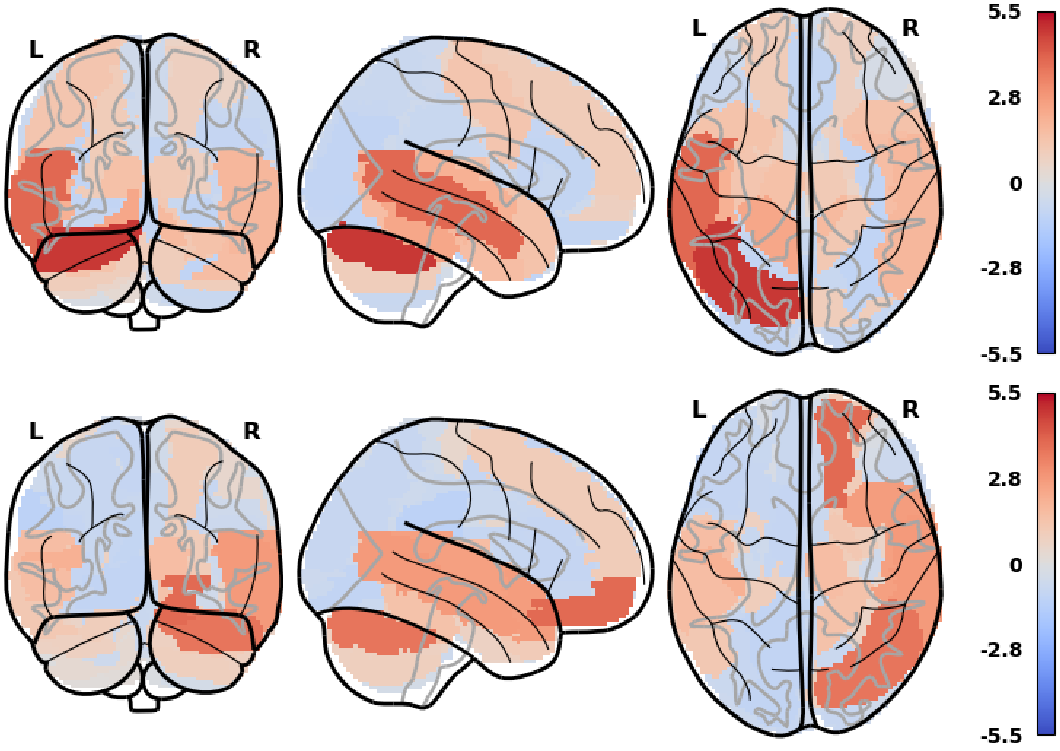

Supp. Figure 14. ResNet importance from Fig. 8 (in the paper) in 4-class encoding (top) and retrieval (bottom). The colour bar shows the z-score of importance.
